# Risk of serious infections with immunosuppressive drugs and glucocorticoids for lupus nephritis: a systematic review and network meta-analysis

**DOI:** 10.1186/s12916-016-0673-8

**Published:** 2016-09-13

**Authors:** Jasvinder A. Singh, Alomgir Hossain, Ahmed Kotb, George Wells

**Affiliations:** 1 Medicine Service, VA Medical Center, Birmingham, AL 35233 USA; 2 Department of Medicine at the School of Medicine, University of Alabama at Birmingham, Birmingham, AL 35294 USA; 3 Division of Epidemiology at the School of Public Health, University of Alabama at Birmingham, Birmingham, AL 35294 USA; 4 Department of Orthopedic Surgery, Mayo Clinic College of Medicine, Rochester, MN 55905 USA; 5 Ottawa Heart Institute and the University of Ottawa, Ottawa, Ontario K1Y 4W7 Canada

**Keywords:** Network meta-analysis, Serious infections, Immunosuppressive drugs, Glucocorticoids, Lupus nephritis, Lupus, Tacrolimus, Cyclophosphamide, Mycophenolate mofetil

## Abstract

**Background:**

To perform a systematic review and network meta-analysis (NMA) to compare the risk of serious infections with immunosuppressive medications and glucocorticoids in lupus nephritis.

**Methods:**

A trained librarian performed two searches: (1) PubMed for all lupus nephritis trials from the end dates for the systematic review for the 2012 American College of Rheumatology (ACR) lupus nephritis treatment guidelines and the 2012 Cochrane Systematic Review on treatments for lupus nephritis, to September 2013; and (2) PubMed and SCOPUS for all lupus trials (excluding lupus nephritis) from inception to February 2014, to obtain additional trials for harms data in any lupus patient. The search was updated to May 2016. Duplicate title/abstract review and duplicate data abstractions by two abstractors independently was performed for all eligible studies, including those studies abstracted for the 2012 ACR lupus nephritis treatment guidelines and the 2012 Cochrane Systematic Review on lupus nephritis treatments. We performed a systematic review and a Bayesian NMA, including randomized controlled trials (RCTs) of immunosuppressive drugs or glucocorticoids in patients with lupus nephritis assessing serious infection risk. Markov chain Monte Carlo methods were used to model 95 % credible intervals (CrI). Sensitivity analyses examined the robustness of estimates.

**Results:**

A total of 32 RCTs with 2611 patients provided data. There were 26 two-arm, five three-arm, and one four-arm trials. We found that tacrolimus was associated with significantly lower risk of serious infections compared to glucocorticoids, cyclophosphamide (CYC), mycophenolate mofetil (MMF), and azathioprine (AZA) with odds ratios (95 % CrI) of 0.33 (0.12–0.88), 0.37 (0.15–0.87), 0.340 (0.18–0.81), and 0.32 (0.12–0.81), respectively. Conversely, CYC low dose (LD), CYC high dose (HD), and HD glucocorticoids were associated with higher odds of serious infections compared to tacrolimus, ranging from 4.84 to 12.83. We also found that MMF followed by AZA (MMF-AZA) was associated with significantly lower risk of serious infections as compared to CYC LD, CYC HD, CYC-AZA, or HD glucocorticoids with odds ratios (95 % CrI) of 0.09 (0.01–0.76), 0.07 (0.01–0.54), 0.14 (0.02–0.71), and 0.03 (0.00–0.56), respectively. Estimates were similar to pair-wise meta-analyses. Sensitivity analyses that varied estimate (odds ratio vs. Peto’s odds ratio), method (random vs. fixed effects model), data (sepsis vs. serious infection data; exclusion of observational studies), treatment grouping (CYC and CYC HD as a combined treatment group vs. separate), made little/no difference to these estimates.

**Conclusions:**

Tacrolimus and MMF-AZA combination were associated with lower risk of serious infections compared to other immunosuppressive drugs or glucocorticoids for lupus nephritis. In conjunction with comparative efficacy data, these data can help patients make informed decisions about treatment options for lupus nephritis.

**PROSPERO registration:**

CRD42016032965

**Electronic supplementary material:**

The online version of this article (doi:10.1186/s12916-016-0673-8) contains supplementary material, which is available to authorized users.

## Background

Lupus nephritis is successfully treated with immunosuppressive drugs and glucocorticoids [1]. The benefits of these lupus treatments are well known with well-documented improved renal survival and function as well as reduced mortality with treatment [2–4]. A key concern for patients with lupus is the risk of infections (including serious infections) with immunosuppressive drugs and glucocorticoids, since both treatments suppress the immune system [5–7]. This concern likely plays a significant role in a patient’s decision regarding whether to take these medications, and how long to continue treatment, sometimes despite a strong physician recommendation.

Whether the risk of serious infections differs by the type of medication in lupus nephritis is unknown and controversial. A meta-analysis of treatments for lupus nephritis showed that the risk of major infection was not higher with cyclophosphamide (CYC) or azathioprine (AZA) plus glucocorticoids versus glucocorticoids alone [8]. In another meta-analysis, mycophenolate mofetil (MMF) was associated with a lower risk of overall infections compared with CYC, with a risk ratio of 0.65 [9]. On the other hand, a more updated and comprehensive 2012 Cochrane systematic review on lupus nephritis treatments showed that MMF was not associated with any significantly different risk of major infections (defined as infections other than herpes zoster) compared to intravenous CYC (the relative risk was 1.11 (95 % confidence interval, 0.74–1.68)) [10, 11]. The findings from these meta-analyses differ due to differences in inclusion criteria, outcome (major infection vs. overall infection) and comparisons. Published meta-analyses, although methodologically sound, suffer from several limitations: (1) comparisons of most drugs were based on a few studies in most cases, thus making them potentially underpowered; (2) some studies included in meta-analyses analyzed major/serious infections while others analyzed all infections, combining these likely led to heterogeneity within such comparisons and also differences between meta-analyses; and (3) indirect comparisons between medications were not performed where head-to-head studies were not available. Lumley et al. [12] described network meta-analysis (NMA) as a way for indirect comparisons of treatments, where head-to-head trials are lacking.

Our aim was to overcome these challenges and conduct a state-of-the-art analysis. We therefore, performed a systematic review, meta-analysis and NMA of all lupus nephritis trials to date to compare the risk of serious infections with immunosuppressive and glucocorticoid treatment in lupus nephritis.

## Methods

### Systematic review methods

We conducted this systematic review and NMA based on the AHRQ recommendations [13] and the Cochrane handbook [14], and reported it according to the PRISMA guidelines [15]. No Institutional Review Board approval was needed since the study included only analyses of published data. The study protocol was registered in PROSPERO, CRD42016032965 (http://www.crd.york.ac.uk/PROSPERO/display_record.asp?ID=CRD42016032965). We used the Cochrane systematic review [10], as well as the systematic review conducted for the American College of Rheumatology guidelines for treatment of lupus nephritis [16], to identify eligible studies. Searches were updated to September 2013 to identify additional studies using PubMed (Additional file 1: Appendix 1A); the search was updated to May 2016. We considered using harms data related to medications from any lupus randomized controlled trial (RCT), not just from lupus nephritis RCTs, by using a search in PubMed and SCOPUS from inception to February 2014 (Additional file 1: Appendix 1B). Examination of the data from this search revealed little additive data for harms for most outcomes of interest (16 RCTs; most had no usable data). Therefore, we determined that the advantage of inclusion of these data was outweighed by the disadvantage of increasing heterogeneity of patient population (from lupus nephritis to lupus). We included RCTs or controlled clinical trials for lupus nephritis that contained the common immunosuppressive drugs including CYC, MMF, AZA, cyclosporine (CSA), tacrolimus (TAC), rituximab (RTX), or glucocorticoids as one of the comparators. Belimumab studies could not be included in this systematic review since these studies excluded patients with active lupus nephritis and a Cochrane systematic review of belimumab for lupus is underway [17]. There were no restrictions with regard to dosage or duration of intervention. We limited our search to the English language. The PICO (Patient, Intervention, Comparator, Outcome) were defined as follows:

- P: Adults 18 years or older, meeting the 1987 American College of Rheumatology Classification criteria for SLE [18] with lupus nephritis.
- I: Interventions were immunosuppressant alone or in combination with other immunosuppressant or biologics (such as RTX) or corticosteroid. Medication doses were categorized as low, standard, or high dose.
- C: Placebo or another immunosuppressant with/without biologic, at any dose of these medications.
- O: Serious infections, defined as any of the following: serious infection, major infection, severe infection, sepsis, or bacterial pneumonia. We refer to these as serious infection from here onwards.

Two trained abstractors independently reviewed abstract and title in duplicate (AO, AB) and selected articles for full PDF download. Two independent abstractors (AO, AB) abstracted all the data in duplicate and entered it directly into Microsoft excel sheets, which were pre-piloted for data abstraction. Another research associate checked the data for accuracy (AM). Studies were abstracted for this analysis if they included adults with lupus nephritis, one of the immunosuppressant drugs or glucocorticoids, and reported serious infection. An adjudicator (JS) resolved any disagreements not resolved by consensus.

Two reviewers (AO, JS) assessed the risk of bias according to the Cochrane risk of bias tool [19], examining the domains of randomization sequence generation, allocation sequence concealment, blinding of participants, personnel and outcome assessors, incomplete outcome data (primary outcome data reporting, dropout rates and reasons for withdrawal, appropriate imputation of missing data, an overall completion rate ≥ 80 %), and selective outcome reporting and other potential threats to validity (considering external validity, e.g., relevant use of co-interventions, bias due to funding source). Each criterion was explicitly judged as follows: Yes = low risk of bias; No = high risk of bias; Unclear = either lack of information or uncertainty about potential for bias. We resolved any disagreements with consensus. We planned funnel plots to assess publication bias.

Pair-wise meta-analysis was conducted when data were sufficiently clinically and statistically homogeneous. We calculated the mean difference (MD) for the continuous and relative risk (RR) or Peto odds ratio (OR) (for rare events), for the dichotomous data. Heterogeneity of data was assessed by using the χ^2^ test, with a *P* value < 0.10 indicating significant heterogeneity, and the I^2^ statistic [20], with values > 50 % indicating substantial heterogeneity.

### NMA methods

We conducted Bayesian mixed treatment comparison (MTC) meta-analyses. We used the WinBUGS software (MRC Biostatistics Unit, Cambridge, UK) to conduct Bayesian MTC meta-analysis using a binomial likelihood model that allows inclusion of multi-arm trials [21, 22]. We used the random-effects network meta-analyses as a conservative approach, since we anticipated some heterogeneity in study populations. We assessed the model fit and chose the model based on the assessment of the deviance information criterion (DIC) and the comparison of residual deviance to the number of unconstrained data points [21, 23].

We assigned vague priors, such as N(0, 100^2^), for basic parameters throughout [21] and informative priors for the variance parameter based on Turner et al. [24]. To ensure convergence was reached, trace plots and the Brooks-Gelman-Rubin statistic were assessed [25]. Three chains were fit in WinBUGS for each analysis, with 40,000 iterations, and a burn-in of 40,000 iterations [25, 26]. We calculated point estimates and 95 % credible intervals (CrI) for OR using the Markov Chain Monte Carlo methods. In order to check the consistency of the NMA results, we conducted the inconsistency analysis and constructed an inconsistency plot, and compared these results with our NMA results based on consistency model [27]. Heterogeneity of data was assessed by using the Tau-squared test, which examines heterogeneity due to study and study–drug interaction (smaller values indicate a better model). There is no specific range for this measure.

Both MTC and traditional meta-analysis require studies to be sufficiently similar in order to pool their results. To further investigate heterogeneity, where warranted, subgroup analyses and meta-regressions [22, 28] were considered. One subgroup analysis we planned was the comparison of estimates before and after 2004 considering potential change in management after the publication of the Contreras et al. [29] study in the *New England Journal of Medicine*. We considered other subgroup analyses by age, gender, race, and prior immunosuppressive therapy for lupus nephritis prior to the current episode, if data were available.

Graphical aids, in the form of network diagrams, were considered for NMA. We compared the NMA results to pair-wise meta-analyses where possible. We performed sensitivity analyses by using OR versus Peto’s OR for estimation. The Grading of Recommendations Assessment, Development and Evaluation (GRADE) approach was used to assess the quality of direct evidence from meta-analysis versus estimates from the NMA, assessing indirectness, imprecision, inconsistency, risk of bias, publication bias, and other factors [30].

Several sensitivity analyses were conducted to test the robustness of main NMA findings, including (1) random versus fixed effects model, (2) the use of sepsis versus serious infection data from Appel et al. [31], (3) the exclusion of data from two observational studies previously included in the 2012 Cochrane review versus inclusion of all studies, and (4) comparing other medications to CYC and CYC high dose as a group versus separately. We conducted sensitivity analyses based on different priors [32] and compared the parameter estimates (i.e., OR (95 % CrI)) and model fit criteria such as DIC and residual deviance with these priors to the main analysis.

## Results

### Study cohort characteristics

**Fig. 1:**
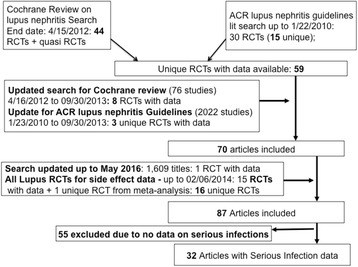
PRISMA flow chart for study selection. A total of 32 studies provided data on serious infections

A total of 32 RCTs with 2611 patients met inclusion criteria and provided data for serious infections (Fig. 1). Detailed study characteristics are provided in Additional file 1: Appendix 2. Six studies examined induction and maintenance phase treatments (19 %), four maintenance phase treatments (13 %), and the rest induction phase treatments (68 %) only. Nine were open-label RCTs and two were quasi-RCTs. Study sample size ranged from 15 to 370. Most studies included patients with diffuse glomerulonephritis and/or membranous glomerulonephritis (Additional file 1: Appendix 2). Studies were mostly single site studies except for nine studies (28 %) that were multicenter. The majority of the patients in the included studies were female. The risk of bias was low for most criteria and unclear for some criteria (Additional file 1: Appendix 3).

**Fig. 2:**
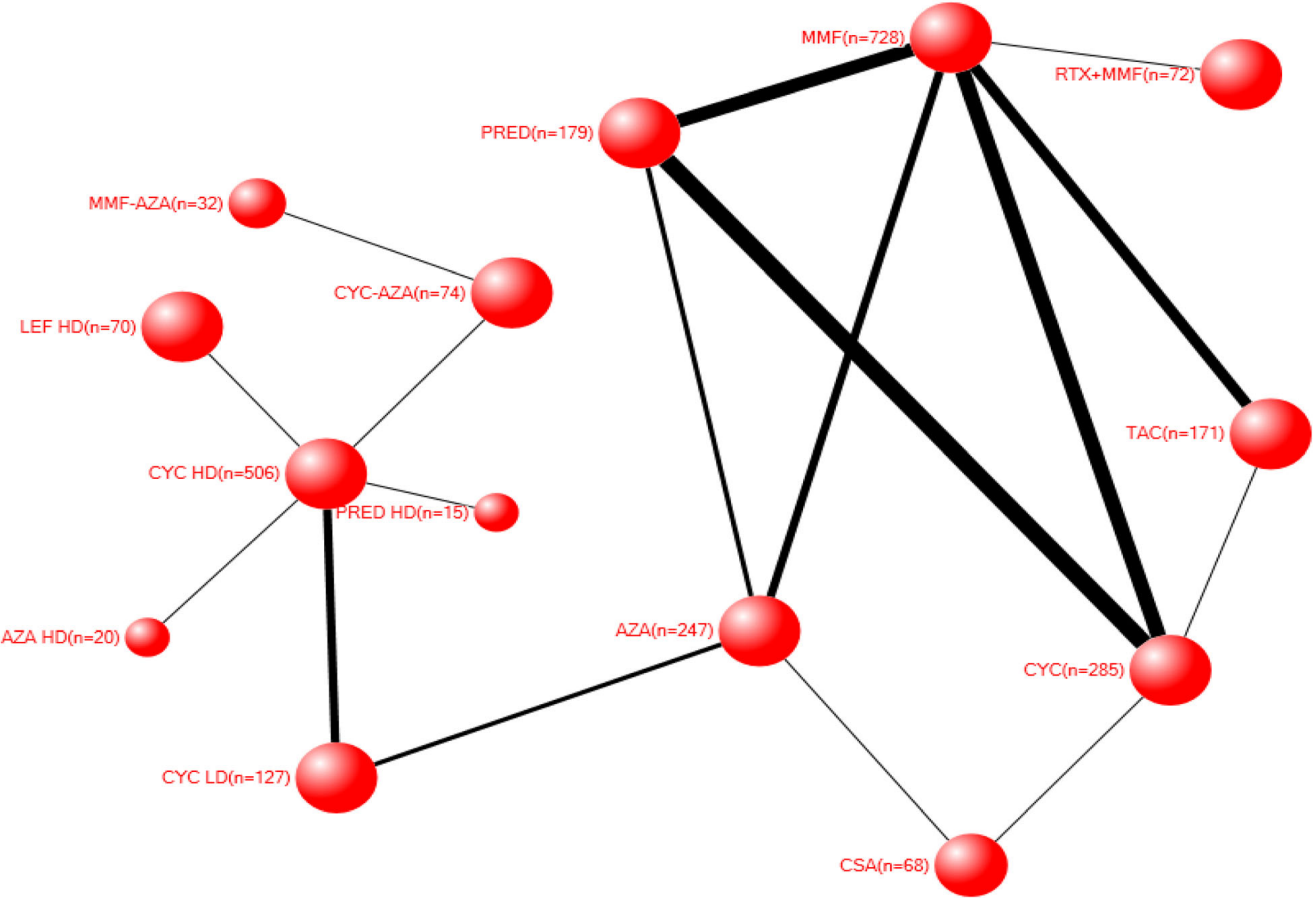
Each node indicates a treatment included in the analysis. The lines represent the direct (head-to-head) comparisons identified in the literature. The surface area of the nodes (represented as circles) represents the number of patients who received the treatment – the larger the size of the circle, the greater the number of patients who received that treatment. For example, there were more patients treated with mycophenolate mofetil or cyclophosphamide (left side of the figure) than tacrolimus or cyclosporine (right side of the figure). Some treatments are noted outside the nodes simply due to longer names that would not fit within the circle size, proportional to the number of patients that received that treatment

The network for serious infections contained multiple treatments as well as sequential and combination regimens (Fig. 2). As expected, most studies compared MMF and high-dose CYC. The network had several direct comparisons of various medications and regimens to each other. These studies included 2611 patients who had 332 serious infections (Additional file 1: Appendix 4). Of these studies, 26 were two-arm, five three-arm, and one four-arm, and these studies included 14 different treatments (Table 1). Six out of 32 studies had at least one arm with zero events. None of the included studies had all arms with zero events (Additional file 1: Appendix 4). The number of serious infections for each of the comparisons (numerator) and the total number of patients available (denominator) is shown in Additional file 1: Appendix 5. For example, for the comparison of glucocorticoids and CYC, 48 serious infections in 335 patients were available and for low- and high-dose, 53 serious infections in 252 patients were available (Additional file 1: Appendix 5).

**Table 1:** Crude rates of serious infections with immunosuppressive drugs or corticosteroids in patients with lupus nephritis

Treatment	#Studies	# Events	# Patients	Aggregate rate, per patient	Minimum rate	Maximum rate
Glucocorticoids	10	30	179	0.181	0.106	0.291
CYC	12	46	285	0.151	0.092	0.240
MMF	12	69	728	0.116	0.072	0.182
AZA	8	45	266	0.184	0.095	0.329
TAC	5	14	171	0.087	0.052	0.141
CSA	3	10	65	0.166	0.091	0.283
CYC LD	4	21	128	0.172	0.114	0.252
HD glucocorticoids	1	4	15	0.267	0.104	0.533
CYC HD	9	60	506	0.131	0.073	0.225
AZA HD	1	2	20	0.100	0.00	1.131
LEF HD	1	3	70	0.043	0.00	0.090
CYC-AZA	3	15	74	0.206	0.118	0.333
MMF-AZA	1	2	32	0.063	0.00	0.146
RTX + MMF	1	12	72	0.167	0.081	0.253

CYC-AZA, CYC followed by AZA

MMF-AZA, MMF followed by AZA

RTX + MMF, RTX combined with MMF

*CYC* cyclophosphamide, *MMF* mycophenolate mofetil, *AZA* azathioprine, *TAC* tacrolimus, *CSA* cyclosporine, *LEF* leflunomide, *HD* high dose, *LD* low dose; when not specified, standard dose should be inferred

### Comparison of immunosuppressive drugs with glucocorticoids

Eleven studies provided data for these comparisons. Crude rates of serious infections with immunosuppressive drugs and glucocorticoids showed the highest crude serious infection rate with high-dose glucocorticoids at 26.7 % (one study only); rates were 15.1 % and 11.6 % with CYC and MMF, respectively (Table 1). Crude rates for serious infections with other treatments are shown in Table 1.

**Fig. 3:**
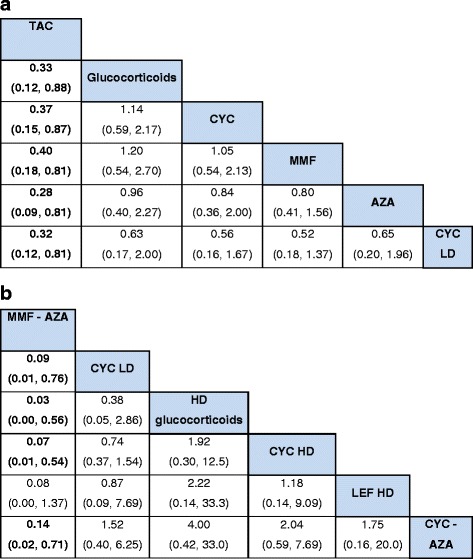
League tables highlight the main findings from the analysis. For each comparison, the random effects model odds ratios (OR) and 95 % credible intervals are provided. The results of the plots are read from top to bottom and left to right. An OR < 1 means that the treatment in the top left is better than the comparator treatment. For example, tacrolimus is better/safer than glucocorticoids (**a**) and mycophenolate mofetil-azathioprine is better/safer than low-dose cyclophosphamide (**b**), since the odds of serious infections are lower in each case than the comparator. Significant results are in bold

We found that tacrolimus was associated with significantly lower risk of serious infections compared to standard-dose corticosteroid, with an OR of 0.33 (95 % CrI, 0.12–0.88) (Table 2). We also found that MMF-AZA (MMF followed by AZA) was associated with significantly lower risk of serious infections as compared to high-dose glucocorticoids, with a relative risk of 0.03 (95 % CrI, 0.00–0.56) (Table 2). Other differences comparing immunosuppressive drugs to glucocorticoids or high-dose glucocorticoids did not reach statistical significance (Fig. 3).

**Table 2:** Serious infection risk in patients with lupus nephritis with various immunosuppressive drugs or corticosteroids showing only the statistically significant results

Treatment	Reference	OR (95 % CrI)	RR (95 % CrI)	RD (95 % Crl)
TAC	Glucocorticoids	0.33 (0.12–0.88)	0.36 (0.14–0.90)	–0.09 (–0.15 to –0.01)
TAC	CYC	0.37 (0.15–0.87)	0.41 (0.17–0.88)	–0.07 (–0.14 to –0.01)
TAC	MMF	0.40 (0.18–0.81)	0.43 (0.21–0.83)	–0.07 (–0.14 to –0.02)
TAC	AZA	0.32 (0.12–0.81)	0.35 (0.14–0.83)	–0.09 (–0.20 to –0.02)
CYC LD	TAC	4.84 (1.48–17.64)	4.00 (1.43–11.47)	0.15 (0.03 to 0.40)
HD glucocorticoids	TAC	12.83 (1.53–119.90)	7.67 (1.47–25.14)	0.35 (0.03 to 0.79)
CYC HD	TAC	6.60 (2.25–20.50)	5.06 (2.03–12.89)	0.20 (0.07 to 0.43)
MMF-AZA	CYC LD	0.09 (0.01–0.76)	0.11 (0.01–0.79)	–0.17 (–0.43 to –0.03)
MMF-AZA	HD glucocorticoids	0.03 (0.00–0.56)	0.06 (0.00–0.61)	–0.37 (–0.82 to –0.04)
MMF-AZA	CYC HD	0.07 (0.01–0.54)	0.09 (0.01–0.60)	–0.22 (–0.46 to –0.06)
MMF-AZA	CYC-AZA	0.14 (0.02–0.71)	0.16 (0.02–0.75)	–0.11 (–0.29 to –0.02)

All odds ratios are statistically significant

High-dose (HD) glucocorticoids were defined as one of the following or a similar regimen: (1) prednisone or methylprednisolone 1 gm/m^2^ qd intravenous × 3 at entry, then one dose intravenous q month for 1 year; (2) prednisone 1 mg/kg po qd with a slow taper up to 1 year or longer taper (or unspecified taper in an occasional case)

Glucocorticoids were defined as one of the following or a similar regimen: (1) prednisone 40 mg po qod for 8 weeks then taper to 10 mg qd within a year; (2) 60 mg qd for 1–3 months reduced to 20 mg/d by 6 months

CYC, low dose (LD): CYC IV 500 mg q 14 d × 6 doses or a similar regimen

CYC: CYC IV 0.5–1.0 gm/m^2^ q 2 month for 1 year or CYC PO 1–4 mg/kg daily for 4 years (standard dose) or a similar regimen

CYC, HD: CYC IV 0.5–1.0 gm/m^2^ q month × 6–9 months, then q3 months for 0.5–4 years or CYC PO 10 mg/kg daily or a similar regimen

MMF-AZA: MMF followed by AZA

CYC-AZA: CYC followed by AZA

*CYC* cyclophosphamide, *MMF* mycophenolate mofetil, *AZA* azathioprine, *TAC* tacrolimus, *HD* high dose, *LD* low dose

### Comparison of immunosuppressive drugs with each other

Twenty-three studies provided data for these comparisons (three studies provided data for both comparisons). Crude rates of serious infections are shown in Table 1, ranging 8.7 % for TAC to 20.6 % with CYC-AZA, for treatments with more than one study. TAC was associated with significantly lower risk of serious infections compared to CYC, MMF, and AZA, with ORs of 0.37, 0.40, and 0.32, respectively (Table 2). We also found that MMF-AZA (MMF followed by AZA) was associated with significantly lower odds of serious infections as compared to low-dose CYC, high-dose CYC, and CYC-AZA with ORs of 0.09, 0.07, and 0.14, respectively (Table 2).

**Fig. 4:**
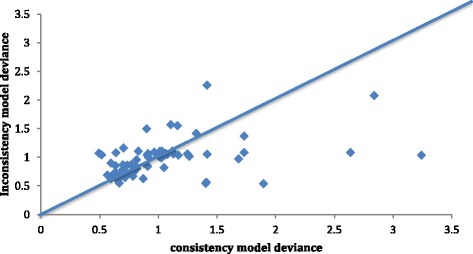
The figure shows the deviances from consistency and the inconsistency models. Consistency model assumes that the evidence derived from direct and indirect estimates should be in agreement. The inconsistency model does not make this assumption. Ideally, all deviances should be 2 or below. There were 71 unconstrained data points. Three data points show deviances of > 2 (extreme right side in the figure). Residual deviances from consistency versus inconsistency models were 72.49 versus 67.73 for 69 data points each. Deviance information criteria was 325.65 versus 325.96, respectively, indicating that the inconsistency model is a slightly better fit for the data than the consistency model. Thus, the model showed some evidence of inconsistency

The staircase diagrams show the ORs for the comparisons of treatments where at least one comparison was significant (Fig. 3). With the exception of the differences discussed in the two sections above, other differences did not reach statistical significance (Fig. 3). In order to check the consistency of the NMA results, we conducted the inconsistency analysis and compared with our consistent NMA results (Fig. 4). Based on this comparison between two models, only three points had a higher than expected posterior mean difference (Fig. 4). Most parameter estimates are very similar between two models. For the overall NMA, the tau (reciprocal of variance between-study; a measure of heterogeneity) was 26.39, and the standard deviation was 0.1949, which is less than 0.5, indicating that heterogeneity is low. We noted some inconsistency of parameter estimates; however, sensitivity analysis that evaluated the results with and without those studies showed that parameter estimates (effects estimates, standard error, etc.) are mostly similar and showed no change on interpretation of the results and conclusions.

### Comparison of NMA results to direct estimates from meta-analyses for main findings

We compared the results from our NMA to traditional pair-wise meta-analyses, where this was possible, TAC versus MMF and TAC versus CYC. The robustness of estimates was indicated by minimal/no differences in estimates between the NMA and the meta-analyses (TAC vs. MMF, 0.40 vs. 0.42; and TAC vs. CYC, 0.37 vs. 0.28, respectively; Additional file 1: Appendix 6). Heterogeneity, as measured by I-squared, was 0 % for TAC versus MMF and 0 % for TAC versus CYC for direct comparisons by meta-analyses. The GRADE quality of evidence was moderate for direct comparison with meta-analyses and moderate for NMA estimates, downgraded for imprecision in both cases.

### Sensitivity analyses

**Fig. 5:**
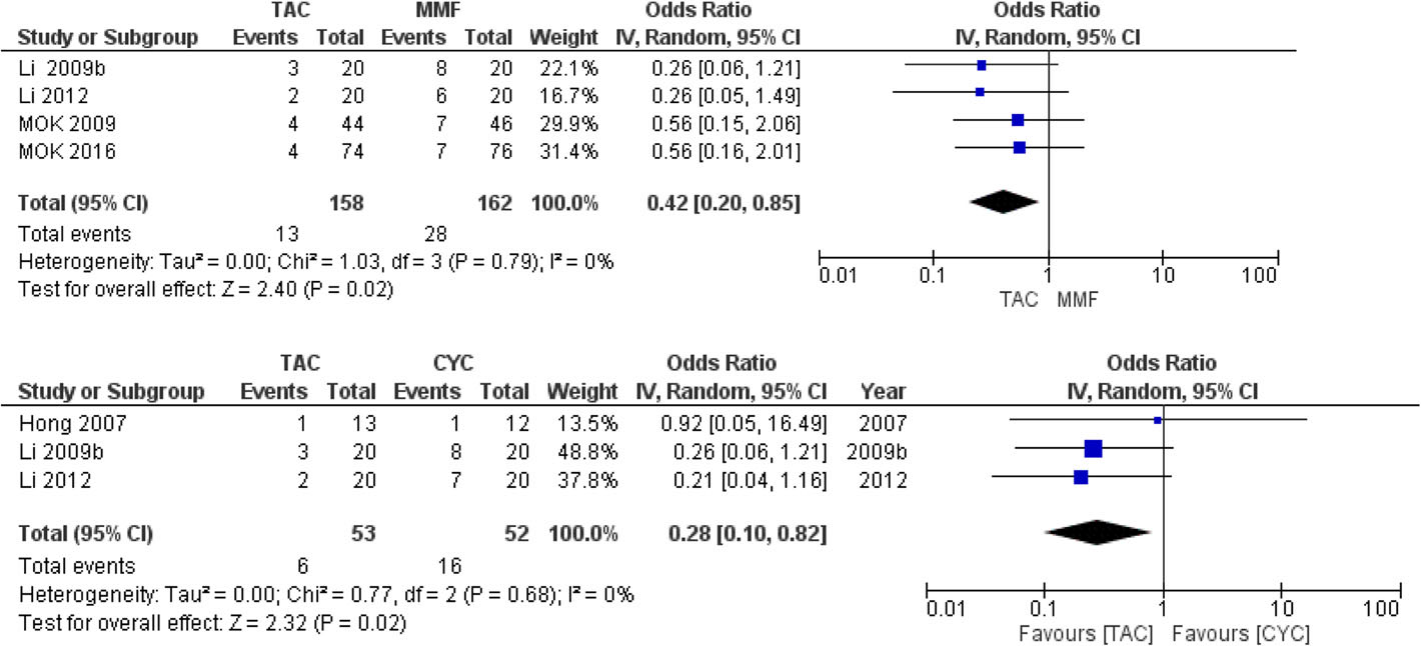
Sensitivity analyses comparing method of estimation, odds ratio versus Peto’s odds ratio for meta-analyses of tacrolimus versus mycophenolate mofetil and tacrolimus versus cyclophosphamide. Same analyses using the Peto Method for rare events

We undertook multiple sensitivity analyses to test the robustness of our study findings. Sensitivity analyses by using the random effects versus fixed effects models (Table 3) or by the type of method used for estimation (OR vs. Peto’s OR) (Fig. 5) revealed no change in interpretation and minimal change in estimates.

**Table 3:** Sensitivity analyses by random versus fixed effects for serious infection risk by comparison of significant estimates between random and fixed effects models

Comparison	Random effects model	Fixed effects model
	Odds ratio (95 % CrI)	Odds ratio (95 % CrI)
TAC vs. glucocorticoids	0.33 (0.12–0.88)	0.33 (0.13–0.82)
TAC vs. CYC	0.37 (0.15–0.87)	0.37 (0.16–0.82)
TAC vs. MMF	0.40 (0.18–0.81)	0.40 (0.20–0.78)
TAC vs. AZA	0.32 (0.12–0.81)	0.32 (0.13–0.77)
CYC LD vs. TAC	4.84 (1.48–17.64)	4.50 (1.43–14.50)
HD glucocorticoids vs. TAC	12.83 (1.53–119.90)	12.22 (1.58–105.70)
CYC HD vs. TAC	6.60 (2.25–20.50)	6.52 (2.38–18.95)
MMF-AZA vs. CYC LD	0.09 (0.01–0.76)	0.09 (0.01–0.74)
MMF-AZA vs. HD glucocorticoids	0.03 (0.00–0.56)	0.21 (0.02–1.79)
MMF-AZA vs. CYC HD	0.07 (0.01–0.54)	0.07 (0.01–0.47)
MMF-AZA vs. CYC-AZA	0.14 (0.02–0.71)	0.14 (0.02–0.65)
Residual deviance	72.49	74.9
Number of unconstrained data points	71	71
Deviance information criteria	325.646	325.51

All odds ratios are statistically significant

Odds ratios from either model are very similar to each other, denoting the robustness of the analyses, regardless of assumptions

*CYC* cyclophosphamide, *MMF* mycophenolate mofetil, *AZA* azathioprine, *TAC* tacrolimus, *HD* high dose, *LD* low dose; when not specified, standard dose should be inferred

Replacing sepsis events (included in serious infection definition) by serious infections in the Appel et al. [31] study revealed slight differences in the significance of MMF-AZA versus low-dose CYC, high-dose glucocorticoids, and high-dose CYC comparisons, i.e., these differences were no longer statistically significant; other comparisons did not change in estimates or significance (Additional file 1: Appendix 7). When we further excluded two studies with observational data [33, 34] from the dataset with serious infection data from Appel et al. [31], we noted no change in estimates or significance except that CYC-AZA versus TAC now became significant, 3.37 (0.75–15.56) versus 14.13 (1.07–220.2), although the confidence intervals were wide (Additional file 1: Appendix 8).

When we combined CYC and high-dose CYC, most associations did not change, except that one MMF-AZA comparison became significant in the combined analyses compared to previously: versus CYC (0.11 (0.01–0.91); Additional file 1: Appendix 9).

Sensitivity analyses based on different priors were performed. The parameter estimates were consistent with these different priors to the main analysis (Additional file 1: Appendix 10). The model fit criteria, such as DIC and residual deviance, were also similar with those priors (Additional file 1: Appendix 10).

### Subgroup analysis

Comparison of studies before and after 2004 was performed considering potential change in management after the publication of the Contreras et al. [29] study in the *New England Journal of Medicine*. For most comparisons, the studies fell into one of the two periods, allowing no meaningful interpretation of results for any time-period effect (Additional file 1: Appendix 11). Since we divided the studies into two time-periods, the network was smaller and 95 % confidence intervals wider and more imprecise with this subgroup analysis. We could not perform subgroup analyses by age, gender, race, and prior immunosuppressive therapy due to lack of enough data presented by these characteristics in the included studies.

## Discussion

In our systematic review and network meta-analyses (NMA), we included a large number of studies and compared the risk of serious infections in patients with lupus nephritis who were treated with immunosuppressive drugs and/or glucocorticoids. We performed a NMA that incorporated indirect and direct comparisons and allowed us to compare several treatments to each other. Importantly, for most of the comparisons, we also had direct comparison trials. We performed several sensitivity analyses and most of these confirmed the robustness of the findings. Several study findings merit further discussion.

We found that TAC was associated with lower risk of serious infections compared to several other immunosuppressive drugs and to standard-dose glucocorticoids. Specifically, compared to CYC, MMF, AZA, and standard-dose glucocorticoids, TAC was associated with one-third the odds of serious infections. High-dose CYC and high-dose glucocorticoids were associated with 7–15 times higher odds of serious infections compared to TAC, providing some evidence for dose-response, with odds going from three-fold for standard dose to 7- to 15-fold for high dose. These are new findings to our knowledge and were robust in multiple sensitivity analyses. Sensitivity analyses we performed, included the following: (1) random or fixed effects model; (2) pair-wise meta-analyses versus NMA; (3) OR versus Peto’s OR for estimation; (4) the use of sepsis versus serious infection data from Appel et al. [31]; (5) exclusion of data from two observational studies previously included in the 2012 Cochrane review versus inclusion of all studies; and (6) comparing to CYC and high-dose CYC in a combined treatment group versus the treatments separately. Thus, the risk of serious infections seems to be less with TAC compared to most comparable immunosuppressive regimens. This finding is similar to that of a large trial in nephrotic syndrome in children, where 4/66 TAC- versus 16/65 CYC-treated patients had serious infections [35]. In a recent systematic review and meta-analysis of TAC for lupus nephritis, TAC (alone or in combination with MMF) was more efficacious than intravenous CYC and as efficacious as MMF for complete or partial renal remission [36]. The review also reported slight mortality advantage with TAC versus controls [36]. Our study finding of lower rate of serious infections, in conjunction with superiority over a previously commonly used therapy (intravenous CYC) might partially explain mortality benefit with TAC.

We did not notice any systematic differences in study populations or the stage of lupus nephritis between trials of TAC versus other immunosuppressive medications. Use of a lower start dose of TAC and titration to achieve trough serum levels may have led to a smaller cumulative TAC dose versus comparators. All included TAC trials were of small sample size and performed in China (non-Caucasian), not unlike several trials for other medications, which were performed internationally.

This information regarding the comparative risk of serious infections with various immunosuppressive drugs in lupus nephritis can be helpful in conjunction with comparative efficacy data that can empower patients to explicitly weigh comparative risks and benefits. These data can be effectively used for informed decision-making by patients with lupus nephritis, in general, but particularly in patients with recurrent infections/serious infections or in those with high-risk comorbidities that increase the risk of serious infections. Compared to Whites, minorities have higher incidence and prevalence of lupus nephritis [37, 38], more severe disease and worse outcomes [39, 40], and more than three times higher mortality [41, 42]. The reasons for these worse outcomes in minorities are not clear, but may be partially related to health access barriers, socioeconomic differences, and higher use of glucocorticoids alone versus immunosuppressives plus glucocorticoids. Future studies need to examine whether the risk difference in serious infections with various medications varies by race/ethnicity.

On the other, we found no significant differences between MMF and CYC for serious infection risk, an important study finding. The OR for serious infection with MMF versus CYC was 0.98 (95 % CrI, 0.50–1.95) in our study, which included 32 studies and was statistically not significant. This is an area of great interest and debate since MMF and CYC are among the most common immunosuppressive drugs used for the treatment of lupus nephritis. In a meta-analysis published in 2007 that included five RCTs with 307 patients [29], MMF was associated with a risk ratio of 0.65 (96 % CI, 0.51–0.82; *P* < 0.001) of overall infections compared with CYC [9]. The most comprehensive study to date, the 2012 Cochrane systematic review that included six studies [31] with infection data for 683 patients (total of 50 studies with 2846 patients included) [10], showed that MMF was not associated with any increase in the risk of major infections (defined as all-cause infections other than herpes zoster) compared to intravenous CYC, and the relative risk was 1.11 (95 % CI, 0.74–1.68) [10]. Our systematic review, meta-analysis, and NMA findings support the earlier finding from the Cochrane systematic review that a difference in the risk of serious infection risk between CYC and MMF was not evident.

However, MMF-AZA was associated with lower odds of serious infections compared to CYC-AZA, high-dose CYC, low-dose CYC, and high-dose glucocorticoids, indicating that some differences may exist between MMF and CYC when followed by AZA. We need data from longer-term studies or studies with a larger sample size. It also remains to be seen whether the EURO-lupus CYC dose (low dose) is a safer option than the regular CYC regimen, especially with regards to the risk of serious infections. This is an important consideration for future studies of harms, since CYC can be given in more than one dose, and most previous analyses have not accounted for CYC dose.

Another important aspect of serious infection risk is the contribution of glucocorticoids to this risk, since they are often used concomitantly with immunosuppressive drugs. A previous meta-analysis of 25 RCTs showed that this risk of major infection did not increase by combining immunosuppressive drugs with glucocorticoids for lupus nephritis treatment, namely, the risk did not differ between CYC or AZA plus glucocorticoids versus glucocorticoids alone [8]. On the other hand the renal-protective benefits of combining immunosuppressive drugs with glucocorticoids for induction therapy have been clearly demonstrated decades ago by an NIH study [43]. Studies that can carefully measure and control for corticosteroid use might help to delineate the contributions they make to serious infection risk when used concomitantly with immunosuppressive drugs. Since our analyses include RCTs, among patients randomized to one of the immunosuppressive drugs, corticosteroid use should be similar except when corticosteroid is used in only one of the treatment arms. Rituximab is an emerging treatment for lupus nephritis with some controversy regarding its efficacy. Our systematic review included only one study with RTX versus placebo in patients receiving MMF and glucocorticoids for lupus nephritis, and no differences were noted in the risk of serious infections versus other medications, including TAC. More data are needed to define its role in the treatment of lupus nephritis.

Study strengths are the use of state-of-the-art systematic review and NMA methodology, the use of large number of trials (and patients) in the analyses, robustness of estimates as demonstrated by no/minimal change in estimates with sensitivity analyses, robustness with models using different priors, and the concordance of findings from NMA with that from traditional pair-wise meta-analyses. The overall quality of evidence for the main findings was moderate, which must be considered while interpreting study results.

Our study has several limitations. Study population heterogeneity between included trials can lead to observations of differences between them, which may be attributable to differences in the types of patients being treated rather than the type of medication being used. In our view, most RCTs were more similar than different, since they enrolled patients with diffuse glomerulonephritis with very few focused on membranous nephropathy and most RCTs were induction or induction/maintenance. While some trials lasted several years, most were 6 months long and the number of patients was less than 100 for the majority of the trials. This leads to small sample sizes and, in cases of rare events such as serious infections, a small number of patients having the outcome. This can lead to suboptimal power to detect all clinically relevant differences between medications, i.e., type II error. We may have missed some differences due to low power. We performed a lot of comparisons, which could have also led to observed significance of some differences just by chance, i.e., type I error. We think this (type I error) is less likely given the rarity of serious infection outcome and our main concern is missing important differences due to the low number of events (type II error). This means that future NMAs in this area will greatly benefit from the addition of new large studies. We limited the search to the English language. Exclusion of studies in non-English language and other databases may have led to missing some pertinent studies. Publication bias assessment was planned (funnel plot), but could not be done due to the lack of treatment pairs with more than 10 studies.

## Conclusions

In summary, we found that tacrolimus was associated with a lower risk of serious infections compared to other immunosuppressives or glucocorticoids. Similar observations were also noted for MMF-AZA. Our systematic review and NMA provides a state-of-the-art analyses of the risk of serious infections with immunosuppressives and glucocorticoids. The overall quality of evidence was moderate. This study provides data that can be used during discussions with patients to better inform them of the comparative risks with various treatment options for lupus nephritis. The results of these analyses will be used in developing a patient decision aid, which will be tested in a randomized trial.

## Additional file

Additional file 1: Additional Files summarizing details of studies includes and senstivity analyses. (DOCX 117 kb)

## Abbreviations

AZA: azathioprine
CrI: credible interval
CSA: cyclosporine
CYC: cyclophosphamide
DIC: deviance information criteria
MMF: mycophenolate mofetil
MTC: mixed treatment comparison
NMA: network meta-analysis
OR: odds ratios
RCTs: randomized controlled trials
RTX: rituximab
TAC: tacrolimus
