# Supplementary material for: Risk of serious infections with immunosuppressive drugs and glucocorticoids for lupus nephritis: a systematic review and network meta-analysis
Source: BMC Med. 2016 Sep 13;14(1):137. doi: 10.1186/s12916-016-0673-8 (PMC5022202; doi:10.1186/s12916-016-0673-8)
Supplement: Additional file 1: — Additional Files summarizing details of studies includes and senstivity analyses. (DOCX 117 kb) [file 12916_2016_673_MOESM1_ESM.docx]

**Appendix**

**Appendix 1.**

**1A.** PubMed search strategy for updating the American College of Rheumatology (ACR) lupus treatment guideline and the Cochrane library searches

(Lupus[text word] OR "Lupus Vulgaris"[MeSH] OR "Lupus Erythematosus, Cutaneous"[MeSH] OR "Lupus Erythematosus, Systemic"[Mesh]) **AND** ("Kidney Diseases"[MeSH] OR nephropath*[text word] OR Transplants[MeSH] OR Transplantation[MesH] OR transplantation[subheading] OR transplant*[text word] OR "Kidney"[Mesh] OR Kidney*[text word] OR Renal*[text word] OR "End Stage Renal Disease"[text word] OR ESRD[text word] OR Glomerulonephr*[text word] OR "GN"[text word] OR "crescentic GN"[text word]) **NOT** ("animals"[MeSH] NOT "humans"[MeSH])

**1B. Second Search strategy to identify any lupus trial for side effects of medications in** PubMed and Scopus databases **(data abstracted, but not used due to scant data and the possibility of heterogeneity in patient population, i.e., lupus nephritis vs. lupus with all other manifestations)**

1. (Lupus Erythematosus, Systemic [mh:noexp] OR “systemic lupus erythematosus” [tw]) AND (Randomized Controlled Trial [pt] OR Randomized Controlled Trials as Topic [mh] OR “randomized controlled” [tw] OR “randomised controlled” [tw]) AND Adult [mh] AND English [la] NOT Nephritis OR Glomerulonephritis

2. (Lupus Erythematosus, Systemic [mh:noexp] OR “systemic lupus erythematosus” [tw]) AND (Randomized Controlled Trial [pt] OR Randomized Controlled Trials as Topic [mh] OR “randomized controlled” [tw] OR “randomised controlled” [tw]) AND English [la] NOT Child OR adolescent OR infant OR newborn OR preschool NOT Nephritis OR Glomerulonephritis

**Appendix 2.** Characteristics of included studies

|  | **Author** | **Year** | **Title** | **Induction/ Maintenance** | **Country** | **Setting** | **Study Design** | **N** | **Treatment group: Male vs. Female or % female**  **or Total N, where sex not provided** |
| --- | --- | --- | --- | --- | --- | --- | --- | --- | --- |
| 1 | Austin | 2009 | Randomized, controlled trial of prednisone, cyclophosphamide, and cyclosporine in lupus membranous nephropathy. J Am Soc Nephrol 2009;20:901–11. | Induction | USA | NIH | RCT | 42 | Glucocorticoids: M/F: 3/12 IV CYC: M/F: 3/12 CSA: M/F: 1/11 |
| 2 | Carette | 1983 | Controlled studies of oral immunosuppressive drugs in lupus nephritis. A long-term follow-up. Ann Intern Med. 1983;99(1):1-8. [148] | Induction and maintenance | USA | NIH | RCT | 53 | Glucocorticoids: M/F: 3/12 AZA: M/F: 2/18 CYC: M/F: 5/13 |
| 3 | Donadio | 1974 | Donadio JVJ, Holley KE, Wagoner RD, Ferguson RH, McDuffie FC. Further observations on the treatment of lupus nephritis with prednisone and combined prednisone and azathioprine. Arthritis & Rheumatism 1974;17(5): 573–81. | Induction | USA | NS | RCT | 16 | M/F: 2/14  Glucocorticoids vs. Glucocorticoids +AZA |
| 4 | Mok | 2001 | Treatment of diffuse proliferative lupus glomerulonephritis: a comparison of two cyclophosphamide-containing regimens. Am J Kidney Dis 2001;38:256–64. | Induction and maintenance | Hong Kong | 2 hospital sites | RCT | 43 | IV pulse CYC, M/F: 1/21;  Pulse CYC followed by AZA, M/F: 1/20 |
| 5 | Dooley | 2011 | Mycophenolate versus azathioprine as maintenance therapy for lupus nephritis. N Engl J Med. 2011;365(20):1886-1895. | Maintenance | United Kingdom | Single center | RCT | 227 | MMF, M/F: 99/116; AZA, M/F: 96/111 |
| 6 | Wang | 2008 | Induction treatment of proliferative lupus nephritis with leflunomide combined with prednisone: a prospective multi-centre observational study. Lupus. 2008;17(7):638-44. [173] | Induction | China | Multicenter | RCT | 110 | LEF, M/F: 10/60;  CYC, M/F: 3/37 |
| 7 | Appel [ALMS study] | 2009 | Appel GB, Contreras G, Dooley MA, Ginzler EM, Isenberg D, Jayne D, et al. Mycophenolate mofetil versus cyclophosphamide for induction treatment of lupus nephritis. Journal of the American Society of Nephrology 2009;20(5):1103–12. | Induction and Maintenance | Multinational | NIH trial, Multicenter | RCT, open-label, parallel-group | 370 | Induction therapy M/F: 57/313; Maintenance therapy M/F: 32/195 |
| 8 | Austin | 1986 | Austin HA, Klippel JH, Balow JE, le Riche NG, Steinberg AD, Plotz PH, et al. Therapy of lupus nephritis. Controlled trial of prednisone and cytotoxic drugs. New England Journal of Medicine 1986;314(10):614–9. | Induction | USA | NIH trials, Multicenter | RCT, open | 107 | M/F: 15/92 |
| 9 | Boumpas | 1992 | Boumpas DT, Austin HA, Vaughn EM, Klippel JH, Steinberg AD, Yarboro CH, et al. Controlled trial of pulse methylprednisolone versus two regimens of pulse cyclophosphamide in severe lupus nephritis. Lancet 1992; 340(8822):741–5. | Induction | USA | NS | RCT | 65 | Pulse glucocorticoids: M/F: 1/24 Pulse CYC: M/F: 3/17 Pulse CYC, then quarterly: M/F: 1/19 |
| 10 | Cade | 1973 | Cade R, Spooner G, Schlein E, Pickering M, DeQuesada A, Holcomb A, et al. Comparison of azathioprine, prednisone, and heparin alone or combined in treating lupus nephritis. Nephron 1973;10(1):37–56. | Induction | USA | Teaching hospital | Quasi-RCT | 54 | HD glucocorticoids: M/F: 3/12 AZA: M/F: 1/12 AZA+Glucocorticoids: M/F: 3/10 AZA+ heparin: M/F: 6/7 |
| 11 | Chan | 2000 | Chan TM, Li FK, Tang CS,Wong RW, Fang GX, Ji YL, et al. Efficacy of mycophenolate mofetil in patients with diffuse proliferative lupus nephritis. Hong Kong-Guangzhou Nephrology Study Group. New England Journal of Medicine 2000;343(16):1156–62. | Induction and maintenance | Hong Kong | Multicenter | RCT | 42 | Glucocorticoids +MMF: M/F: 6/26 Glucocorticoids+CYC, then Glucocorticoids+AZA: M/F: 4/26 |
| 12 | Contreras | 2002 | Contreras G, Pardo V, Leclercq B, Lenz O, Tozman E,  O’Nan P, et al. Sequential therapies for proliferative lupus nephritis. New England Journal of Medicine 2004;350(10): 971–80. [MEDLINE: 14999109] &  Contreras G, Pardo V, Leclercq B, Gomez E, Reich J, O’Nan P, et al. Maintenance therapy for proliferative forms of lupus nephritis: a randomized clinical trial comparing quarterly intravenous cyclophosphamide (IVCY) versus oral mycophenolate mofetil (MMF) or azathioprine (AZA) [abstract]. Journal of the American Society of Nephrology 2002;13(Program & Abstracts):15A | Maintenance | USA | Single center | RCT, Open-label | 59 | IV CYC: M/F: 1/19 AZA: M/F: 2/18 MMF: M/F: 1/19 |
| 13 | Donadio | 1978 | Donadio JV, Holley KE, Ferguson RH, Ilstrup DM. Treatment of diffuse proliferative lupus nephritis with prednisone and combined prednisone and cyclophosphamide. New England Journal of Medicine 1978; 299(21):1151–5. | Induction | USA | Single center | RCT, Open-label | 26 | Glucocorticoids: M/F: 4/22 Glucocorticoids +CYC: M/F: 5/19 |
| 14 | El-Shafey | 2010 | El Shafey EM, Abdou SH, ShareefMM. Is mofetil superior to pulse intravenous cyclophosphamide for induction therapy of proliferative lupus nephritis in Egyptian patients. Clinical & Experimental Nephrology 2010;14(3):214–21. | Induction | Egypt | Single center | RCT, open-label | 47 | MMF: M/F: 1/23 Pulse IV CYC: M/F: 1/22 |
| 15 | Ginzler | 2005 | Ginzler EM, Dooley MA, Aranow C, Kim MY, Buyon J, Merrill JT, et al. Mycophenolate mofetil or intravenous cyclophosphamide for lupus nephritis. New England Journal of Medicine 2005;353(21):2219–28. | Induction | USA | Single Center | RCT, open-label, non-inferiority | 140 | MMF: M/F: 10/61 CYC: M/F: 4/65 |
| 16 | Gourley | 1996 | Gourley MF, Austin HA, Scott D, Yarboro CH, Vaughan EM, Muir J, et al. Methylprednisolone and cyclophosphamide, alone or in combination, in patients with lupus nephritis. A randomized, controlled trial. Annals of Internal Medicine 1996;125(7):549–57. | Induction | USA | Single Center | RCT | 82 | Glucocorticoids: M/F: 5/22 CYC: M/F: 6/21 CYC +Glucocorticoids: M/F: 3/25 |
| 17 | Hahn | 1975 | Hahn BH, Kantor OS, Osterland CK. Azathioprine plus prednisone compared with prednisone alone in the treatment of systemic lupus erythematosus. Report of a prospective controlled trial in 24 patients. Annals of Internal Medicine 1975;83(5):597–605. | Induction | USA | Single Center | RCT | 20 | Glucocorticoids: M/F: 2/11 AZA: M/F: 2/9 |
| 18 | Hong | 2007 | Hong R, Haijin Y, Xianglin W, Cuilan H, Nan C. A preliminary study of tacrolimus versus cyclophosphamide in patients with diffuse proliferative lupus nephritis [abstract]. Nephrology Dialysis Transplantation 2007;22(Suppl 6): vi276. | Induction | China | NS | RCT | 25 | Not available |
| 19 | Houssiau (Euro-Lupus Nephritis Trial) | 2002 | Houssiau FA, Vasconcelos C, D’Cruz D, Sebastiani GD, Garrido Ed Ede R, Danieli MG, et al. Immunosuppressive therapy in lupus nephritis: the Euro-Lupus Nephritis Trial, a randomized trial of low-dose versus high-dose intravenous cyclophosphamide. Arthritis & Rheumatism 2002;46(8): 2121–31. | Induction and maintenance | European | Multicenter | RCT | 90 | High-dose IV CYC followed by AZA: M/F: 3/43 Low-dose IV CYC followed by AZA M/F: 3/41 |
| 20 | Li | 2009b | Li X, Ren H, Zhang W, Xu Y, Shen P, Zhang Q, et al. Induction therapies for proliferative lupus nephritis: mycophenolate mofetil, tacrolimus and intravenous cyclophosphamide [abstract]. Journal of the American Society of Nephrology 2009;20:391A. | Induction | China | NS | RCT, Open-label | 60 | MMF: M/F: 3/17  TAC: M/F: 3/17  CYC: M/F: 2/18 |
| 21 | LUNAR Study | 2012 | Rovin BH, Furie R, Latinis K, Looney RJ, Fervenza FC, Sanchez-Guerrero J, et al. Efficacy and safety of rituximab in patients with active proliferative lupus nephritis: the Lupus Nephritis Assessment with Rituximab study. Arthritis & Rheumatism. 2012;64(4):1215-26 &  Rovin BH, Appel G, Furie R, Looney J, Latinis K, Fervenza FC, et al. Efficacy and safety of rituximab (RTX) in subjects with proliferative lupus nephritis (LN): results from the randomized, double-blind phase III LUNAR study [abstract]. Journal of the American Society of Nephrology 2009;20:77A. | Induction | Multinational | NIH trials, multicenter | RCT | 144 | Std therapy +Placebo: M/F: 5/67  Std therapy +RTX: M/F: 9/63 |
| 22 | Mitwalli | 2011 | Mitwalli AH, Al Wakeel JS, Hurraib S, Aisha A, Al Suwaida A, Alam A, et al. Comparison of high and low dose of cyclophosphamide in lupus nephritis patients: a long-term randomized controlled trial. Saudi Journal of Kidney Diseases & Transplantation 2011;22(5):935–40. | Induction and maintenance | Saudi Arabia | Single Center | RCT | 117 | CYC HD: M/F: 12/61 CYC LD: M/F: 5/39 |
| 23 | Mok | 2009 | Mok CC, Ying SK, Tong KH, Siu YP, To CH, Yim CW, et al. Mycophenolate mofetil versus tacrolimus for active lupus nephritis: an extended observation of a randomized controlled trial [abstract]. Annals of the Rheumatic Diseases 2009;68(Suppl 3):246. | Induction | Hong Kong, China | NS | RCT | 109 | M/F: 11/98  MMF vs. TAC |
| 24 | Moroni | 2004 | Moroni G, Doria A, Mosca M, Ferraccioli G, Todesco S, Manno C, et al. A randomized trial comparing cyclosporine versus azathioprine for maintenance therapy in diffuse lupus nephritis [abstract]. Journal of the American Society of Nephrology 2004;15(Oct):121A. | Maintenance | Italy | Multicenter | RCT | 69 | CSA: M/F: 3/33 AZA: M/F: 4/29 |
| 25 | Li | 2012 | Mycophenolate mofetil or tacrolimus compared with intravenous cyclophosphamide in the induction treatment for active lupus nephritis. Nephrology Dialysis Transplantation. 2012;27(4):1467-1472. | Induction | China | Single center | RCT | 60 | MMF: M/F: 3/17 Tacrolimus: M/F: 3/17 IV CYC: M/F: 2/18 |
| 26 | Ong | 2005 | Ong LM, Hooi LS, LimTO, Goh BL, AhmadG, Ghazalli R, et al. Randomized controlled trial of pulse intravenous cyclophosphamide versus mycophenolate mofetil in the induction therapy of proliferative lupus nephritis. Nephrology 2005;10(5):504–10. | Induction | Malaysia | Multicenter | RCT, open-label | 54 | MMF: M/F: 3/23 IV CYC: M/F: 4/15 |
| 27 | Sabry | 2009 | Sabry A, Sheashaa H, Mahmoud K, Elhuusieni A, El Dahshan K. A comparative study of two intensified pulse cyclophosphamide remission-inducing regimen for diffuse proliferative lupus nephritis: an Egyptian experience [abstract]. Nephrology Dialysis Transplantation 2007;22 (Suppl 6):vi28. | Induction | Egypt | Single Center | Quasi-RCT | 46 | CYC, HD: M/F: 4/22 CYC, LD: M/F: 2/18 |
| 28 | Sesso | 1994 | Sesso R, Monteiro M, Sato E, Kirsztajn G, Silva L, Ajzen H. A controlled trial of pulse cyclophosphamide versus pulse methylprednisolone in severe lupus nephritis. Lupus 1994;3(2):107–12. | Induction | Brazil | Single Center | RCT | 29 | IV CYC: M/F: 2/12 IV Glucocorticoids: M/F: 2/13 |
| 29 | Steinberg | 1971 | Steinberg AD, Kaltreider HB, Staples PJ, Goetzl EJ, Talal N, Decker JL. Cyclophosphamide in lupus nephritis: a controlled trial. Annals of Internal Medicine 1971;75(2): 165–71. | Induction | USA | Single Center | RCT | 15 | IV CYC +Glucocorticoids: M/F: 0/7 Placebo +Glucocorticoids: M/F: 0/6 |
| 30 | MAINTAIN Nephritis Study | 2010 | Houssiau FA, D’CruzD, Sangle S, Remy P, Vasconcelos C, Petrovic R, et al. Azathioprine versus mycophenolate mofetil for long-term immunosuppression in lupus nephritis: results from the MAINTAIN Nephritis Trial. Annals of the Rheumatic Diseases 2010;69(12):2083–9. [MEDLINE: 20833738] | Maintenance | European | Multicenter | RCT | 105 | AZA: M/F: 4/48  MMF: M/F: 5/48 |
| 31 | Lui | 1997 | Lui SF, Cheng IKP, Tong KL, Li CS, Wong KC, Chang DT, Sang WK, Chau KF. Treatment of type iv lupus nephritis (LN) - comparison of 2 triple therapy regimens: cyclosporin a (CSA), prednisolone (PRED), azathioprine (AZA) vs. oral cyclophosphamide (POCP), prednisolone, azathioprine [abstract]. Nephrology 1997;3(Suppl 1):S476 | Induction | Hong Kong | NS | RCT | 34 | Not available  CSA +Glucocorticoids +AZA vs.  PO CYC +Glucocorticoids +AZA |
| 32 | Mok | 2016 | Mok CC, Ying KY, Yim CW, Siu YP, Tong KH, To CH, et al. Tacrolimus versus mycophenolate mofetil for induction therapy of lupus nephritis: a randomised controlled trial and long-term follow-up. Annals of the rheumatic diseases. 2016;75(1):30-6 | Induction | Hong Kong | NS | Open RCT | 150 | MMF: M/F: 8/68  TAC: M/F: 4/70 |

NS, not specified; RCT, randomized controlled trial; Std, standard; IV, intravenous; PO, oral; NIH, National Institutes of Health

M, male; F, female

CYC, cyclophosphamide; MMF, mycophenolate mofetil; AZA, azathioprine; TAC, tacrolimus; CSA, cyclosporine; LEF, leflunomide; PLASMA, plasmapharesis

HD, high dose; LD, low dose; SD, standard dose; when dose is not specified, standard dose should be inferred.

**Appendix 3.** Risk of bias of included studies according to the Cochrane Risk of Bias tool^1^

| Author | Year | Randomization | Allocation Concealment | Blinding of Assessor and/or physician (for assessment of objective outcomes) | Blinding of Participants (for assessment of subjective outcomes) | Intention to Treat | Free of Selective Reporting | Source of Funding |
| --- | --- | --- | --- | --- | --- | --- | --- | --- |
| Austin | 2009 | Low risk | Low risk | High risk | High risk | Unclear risk | Unclear risk | Unclear risk |
| Carette | 1983 | Unclear risk | Unclear risk | High risk | High risk | Low risk | High risk | Low risk |
| Donadio | 1974 | Low risk | Unclear risk | Low risk | Low risk | Unclear risk | High risk | Low risk |
| Mok | 2001 | N/A | N/A | N/A | N/A | N/A | N/A | N/A |
| Dooley | 2011 | Unclear risk | Unclear risk | Unclear risk | Unclear risk | High risk | Unclear risk | High risk |
| Wang | 2008 | N/A | N/A | N/A | N/A | N/A | N/A | N/A |
| Appel (ALMS study) | 2009 | Low risk | Low risk | Low risk | Unclear risk | Low risk | Low risk | Unclear risk |
| Austin | 1986 | Low risk | Unclear risk | Unclear risk | Unclear risk | Unclear risk | Unclear risk | Unclear risk |
| Boumpas | 1992 | Unclear risk | Low risk | Low risk | Low risk | Low risk | Low risk | Unclear risk |
| Cade | 1973 | High risk | Unclear risk | Low risk | Low risk | Unclear risk | Unclear risk | Unclear risk |
| Chan | 2000 | Low risk | Unclear risk | Low risk | Low risk | Low risk | Low risk | Unclear risk |
| Contreras | 2002 | Low risk | Low risk | High risk | High risk | Low risk | Low risk | Unclear risk |
| Donadio | 1978 | Low risk | Unclear risk | Low risk | Low risk | Unclear risk | Unclear risk | Unclear risk |
| El-Shafey | 2010 | Low risk | Unclear risk | Low risk | Low risk | Low risk | Low risk | Low risk |
| Ginzler | 2005 | Low risk | Low risk | Low risk | Low risk | Low risk | Low risk | Unclear risk |
| Gourley | 1996 | Low risk | Low risk | Low risk | Low risk | Low risk | Low risk | Low risk |
| Hahn | 1975 | Low risk | Low risk | Low risk | Low risk | Low risk | Low risk | Low risk |
| Hong | 2007 | Unclear risk | Unclear risk | Unclear risk | Unclear risk | Unclear risk | Unclear risk | Unclear risk |
| Houssiau | 2002 | Low risk | Unclear risk | Low risk | Low risk | Low risk | Low risk | Low risk |
| Li | 2009b | Unclear risk | Unclear risk | Low risk | Low risk | Low risk | Low risk | Low risk |
| LUNAR Study | 2012 | Low risk | Unclear risk | Low risk | Low risk | Low risk | Low risk | Unclear risk |
| Mitwalli | 2011 | Unclear risk | Unclear risk | Unclear risk | Unclear risk | Unclear risk | Unclear risk | Unclear risk |
| Mok | 2009 | Unclear risk | Unclear risk | Unclear risk | Unclear risk | Unclear risk | Unclear risk | Unclear risk |
| Moroni | 2004 | Low risk | Low risk | Low risk | Low risk | Low risk | Low risk | Unclear risk |
| Li | 2012 | Unclear risk | Unclear risk | High risk | High risk | High risk | Unclear risk | Low risk |
| Ong | 2005 | Low risk | Low risk | Low risk | Low risk | Low risk | Low risk | Unclear risk |
| Sabry | 2009 | High risk | High risk | Low risk | Low risk | Low risk | Low risk | Low risk |
| Sesso | 1994 | Unclear risk | Unclear risk | High risk | Low risk | Low risk | Unclear risk | Low risk |
| Steinberg | 1971 | Low risk | Low risk | Low risk | Low risk | Low risk | Low risk | Unclear risk |
| MAINTAIN Study | 2010 | Low risk | Unclear risk | Low risk | Low risk | Low risk | Low risk | Low risk |
| Lui | 1997 | Unclear risk | Unclear risk | Unclear risk | Unclear risk | Unclear risk | Unclear risk | Unclear risk |
| Mok | 2016 | Low risk | Low risk | High risk | High risk | Low risk | Low risk | Low risk |

N/A= not applicable

Mok 2001 and Wang 2008 were observational studies used in the Cochrane Review, therefore risk of bias could not be assessed for these studies.

^1^Higgins JP, Altman DG, Sterne JA. Chapter 8: Assessing risk of bias in included studies. Cochrane Handbook for Systematic Reviews of Interventions Version 5.1.0 (updated March 2011). The Cochrane Collaboration, 2011. Available from <http://www.cochrane-handbook.org>. In: Higgins JP, Green S, eds2011.

**Appendix 4.** Network Characteristics

| **Characteristic** | **Number** |
| --- | --- |
| **Number of Interventions** | 14 |
| **Number of Studies** | 32 |
| **Total Number of Patients in Network** | 2,610 |
| **Total Number of Events in Network** | 332 |
| **Total Possible Pairwise Comparisons** | 91 |
| **Total Number Pairwise Comparisons With Direct Data** | 24 |
| **Number of Two-arm Studies** | 26 |
| **Number of Multi-Arms Studies** | 6 |
| **Number of Studies With No Zero Events** | 26 |
| **Number of Studies With At Least One Zero Event (in at least one study treatment arm)** | 6 |
| **Number of Studies with All Zero Events (in all study treatment arms)** | 0 |

**Appendix 5.** Characteristics of studies providing Direct Comparison of treatments for serious infection outcome

|  | | |  |
| --- | --- | --- | --- |
| **Comparison** | **# Studies** | **# Patients** | **# Events** |
| Glucocorticoids vs. CYC | 7 | 335 | 48 |
| Glucocorticoids vs. AZA | 4 | 115 | 27 |
| Glucocorticoids vs. CYC-AZA | 1 | 50 | 10 |
| CYC vs. AZA | 1 | 57 | 7 |
| CYC vs. CYC-AZA | 1 | 60 | 8 |
| AZA vs. CYC-AZA | 1 | 41 | 5 |
| Glucocorticoids vs. CSA | 1 | 27 | 3 |
| CYC vs. CSA | 2 | 61 | 4 |
| CYC vs. MMF | 3 | 127 | 33 |
| CYC vs. TAC | 3 | 105 | 22 |
| MMF vs. TAC | 4 | 320 | 41 |
| MMF vs. CYC HD | 3 | 564 | 24 |
| MMF vs. AZA | 3 | 370 | 37 |
| MMF vs. CYC LD | 1 | 40 | 5 |
| AZA vs. CYC LD | 1 | 39 | 5 |
| MMF vs. RTX+MMF | 1 | 144 | 24 |
| AZA vs. CSA | 1 | 69 | 21 |
| CYC LD vs. CYC HD | 3 | 252 | 53 |
| HD glucocorticoids vs. CYC HD | 1 | 33 | 7 |
| HD glucocorticoids vs. AZA HD | 1 | 35 | 6 |
| CYC HD vs. AZA HD | 1 | 38 | 5 |
| CYC HD vs. CYC-AZA | 1 | 43 | 6 |
| CYC HD vs. LEF HD | 1 | 110 | 5 |
| CYC-AZA vs. MMF-AZA | 1 | 63 | 11 |

**Appendix 6.** Comparing evidence from the Network meta-analysis with evidence obtained from the only possible pairwise meta-analysis conducted

| **Comparison** | **Odds ratios and 95% Confidence Intervals from Direct comparison only** | **Odds ratios and 95% Credible Intervals from the Network Meta-Analysis** |
| --- | --- | --- |
| TAC versus MMF | **0.42 (0.20, 0.85)^1^** | **0.40 (0.18, 0.81)^3^** |
| TAC versus CYC | **0.28 (0.10, 0.80)^2^** | **0.37(0.15, 0.87))^4^** |
| **Note**: The I^2^ = 0% in both treatment comparisons. Please refer to the forest plots below for the number of studies, patients and events in each comparison.  9 comparisons out of a possible 24 could be meta-analyzed. Out of those 9, only 2 were significant from the network analysis. The pairwise meta-analyses of these two comparisons were conducted and are reported here in comparison with the estimates from the network analysis. The table shows comparison of estimates from pairwise meta-analysis compared to NMA. The plots show results using using odds ratio (first two plots) or the Peto’s odds ratio (used for rare outcomes), showing consistency in estimates.  **All odds ratios in bold are statistically significant with a p-value <0.05**  ^1^I-squared = 0%; heterogeneity chi-square = 1.03 and p-value = 0.79; ; Quality of evidence as judged based on the Grading of Recommendations Assessment, Development and Evaluation (GRADE) approach, ⊕⊕⊕O moderate [downgraded for imprecision]  ^2^I-squared = 0%; heterogeneity chi-square = 0.77 and p-value = 0.68; Quality of evidence, ⊕⊕⊕O moderate [downgraded for imprecision]  ^3^ Quality of evidence, ⊕⊕⊕O moderate [downgraded for imprecision]  ^4^ Quality of evidence, ⊕⊕⊕O moderate [downgraded for imprecision] | | |

**Appendix 7.** Sensitivity analyses: replacement of sepsis data from Appel et al. with serious infection data

|  |  | **Old Main Analyses using**  **Apple (Sepsis data)** | | | **Sensitivity analysis using**  **Appel (serious infection data)** | | |
| --- | --- | --- | --- | --- | --- | --- | --- |
| **Treatment** | **Reference** | **OR (95% CrI)** | **RR (95% CrI)** | **RD % (95% Crl)** | **OR (95% CrI)** | **RR (95% CrI)** | **RD % (95% Crl)** |
| TAC | Glucocorticoids | **0.33 (0.12, 0.88)** | **0.36 (0.14, 0.90)** | **-0.09 (-0.15, -0.01)** | **0.36 (0.12,0.98)** | **0.39 (0.14,0.98)** | **-0.08 (-0.15,0.00)** |
| TAC | CYC | **0.37 (0.15, 0.87)** | **0.41 (0.17, 0.88)** | **--0.07 (-0.14, -0.01)** | **0.40 (0.16,0.98)** | **0.44 (0.18,0.98)** | **-0.07 (-0.14,0.00)** |
| TAC | MMF | **0.40 (0.18, 0.81)** | **0.43 (0.21, 0.83)** | **-0.07 (-0.14, -0.02)** | **0.38 (0.17,0.80)** | **0.41 (0.19,0.82)** | **-0.07 (-0.16,-0.02)** |
| TAC | AZA | **0.32 (0.12, 0.81)** | **0.35 (0.14, 0.83)** | **-0.09 (-0.20, -0.02)** | **0.32 (0.11,0.87)** | **0.36 (0.13,0.88)** | **-0.10 (-0.22,-0.01)** |
|  |  |  |  |  |  |  |  |
| CYC LD | TAC | **4.84 (1.48, 17.64)** | **4.00(1.43, 11.47)** | **0.15 (0.03, 0.40)** | **4.18 (1.27,17.89)** | **3.51 (1.24,11.36)** | **0.13 (0.02,0.41)** |
| HD Glucocorticoids | TAC | **12.83 (1.53, 119.90)** | **7.67(1.47, 25.14)** | **0.35 (0.03, 0.79)** | **9.93 (1.16,108.0)** | **6.39 (1.15,23.20)** | **0.30 (0.01,0.79)** |
| CYC HD | TAC | **6.60 (2.25, 20.50)** | **5.06 (2.03, 12.89)** | **0.20 (0.07, 0.43)** | **5.23 (1.94,17.75)** | **4.18 (1.79,11.59)** | **0.17 (0.05,0.40)** |
|  |  |  |  |  |  |  |  |
| MMF-AZA | CYC-LD | **0.09 (0.01, 0.76)** | **0.11 (0.01, 0.79)** | **-0.17 (-0.43, -0.03)** | 0.38 (0.06,1.86) | 0.44 (0.09,1.69) | -0.10 (-0.38,0.08) |
| MMF-AZA | HD Glucocorticoids | **0.03 (0.00, 0.56)** | **0.06 (0.00, 0.61)** | **-0.37 (-0.82, -0.04)** | 0.16 (0.01,1.78) | 0.24 (0.04,1.64) | -0.27 (-0.76,0.06) |
| MMF-AZA | CYC HD | **0.07 (0.01, 0.54)** | **0.09 (0.01, 0.60)** | **-0.22 (-0.46, -0.06)** | 0.30 (0.06,1.31) | 0.37 (0.08,1.24) | -0.14 (-0.37,0.04) |
| MMF-AZA | CYC-AZA | **0.14 (0.02, 0.71)** | **0.16 (0.02, 0.75)** | **-0.11 (-0.29, -0.02)** | **0.19 (0.04,0.80)** | **0.28 (0.08,0.83)** | **-0.22 (-0.58,-0.02)** |
| **All odds ratios in bold are statistically significant with a p-value <0.05**  High dose (HD) glucocorticoids were defined as one of the following or a similar regimen: (1) prednisone or methylprednisolone 1 gm/m^2^ qd intravenous x 3 at entry, then one dose intravenous q month for 1 year; (2) prednisone 1 mg/kg po qd with a slow taper up to 1 year or longer taper (or unspecified taper in an occasional case).  Glucocorticoids were defined as one of the following or a similar regimen: (1) prednisone 40 mg po qod for 8 weeks then taper to 10 mg qd within a year; (2) 60 mg qd for 1-3 months reduced to 20 mg/d by 6 months  CYC, low dose (LD): CYC IV 500 mg q 14 d x 6 doses or a similar regimen  CYC: CYC IV 0.5-1.0 gm/m^2^ q 2month for 1 year or CYC PO 1-4 mg/kg daily for 4 yrs (standard dose (SD)) or a similar regimen  CYC, HD: CYC IV 0.5-1.0 gm/m^2^ q month x 6-9 months, then q3 months for 0.5-4 years or CYC PO 10 mg/kg daily or a similar regimen  MMF-AZA: MMF followed by AZA  CYC-AZA: CYC followed by AZA  OR, odds ratio; RR, relative risk ratio; RD, risk difference; CrI, credible interval | | | | | | | |

**Appendix 8.** Sensitivity analyses with the exclusion of studies with observational data

|  |  | **New Analyses* with**  **Observational Studies Removed** | | | **Main Analyses (serious infection data only)**** | | |
| --- | --- | --- | --- | --- | --- | --- | --- |
| **Treatment** | **Reference** | **OR (95% CrI)** | **RR (95% CrI)** | **RD (95% Crl)** | **OR (95% CrI)** | **RR (95% CrI)** | **RD (95% Crl)** |
| TAC | Glucocorticoids | **0.35 (0.12,0.98)** | **0.38 (0.14,0.98)** | **-0.08 (-0.15,0.00)** | **0.36 (0.12,0.98)** | **0.39 (0.14,0.98)** | **-0.08 (-0.15,0.00)** |
| TAC | CYC | **0.39 (0.15,0.96)** | **0.42 (0.17,0.97)** | **-0.07 (-0.14,0.00)** | **0.40 (0.16,0.98)** | **0.44 (0.18,0.98)** | **-0.07 (-0.14,0.00)** |
| TAC | MMF | **0.38 (0.17,0.82)** | **0.42 (0.19,0.84)** | **-0.07 (-0.15,-0.02)** | **0.38 (0.17,0.80)** | **0.41 (0.19,0.82)** | **-0.07 (-0.16,-0.02)** |
| TAC | AZA | **0.32 (0.11,0.86)** | **0.35 (0.13,0.88)** | **-0.09 (-0.21,-0.01)** | **0.32 (0.11,0.87)** | **-0.10 (-0.22,-0.01)** | **-0.10 (-0.22,-0.01)** |
|  |  |  |  |  |  |  |  |
| CYC LD | TAC | **3.98 (1.19,17.68)** | **3.40 (1.17,11.30)** | **0.12 (0.01,0.40)** | **4.18 (1.27,17.89)** | **3.51 (1.24,11.36)** | **0.13 (0.02,0.41)** |
| HD glucocorticoids | TAC | **9.70 (1.13,103.80)** | **6.37 (1.12,23.43)** | **0.29 (0.01,0.78)** | **9.93 (1.16,108.00)** | **6.39 (1.15,23.20)** | **0.30 (0.01,0.79)** |
| CYC HD | TAC | **4.99 (1.80,17.48)** | **4.07 (1.68,11.44)** | **0.16 (0.05,0.40)** | **5.23 (1.94,17.75)** | **4.18 (1.79,11.59)** | **0.17 (0.05,0.40)** |
|  |  |  |  |  |  |  |  |
| MMF-AZA | CYC-LD | 0.50 (0.06,2.94) | 0.56 (0.09,2.42) | -0.07 (-0.37,0.15) | 0.38 (0.06,1.86) | 0.44 (0.09,1.69) | -0.10 (-0.38,0.08) |
| MMF-AZA | HD glucocorticoids | 0.20 (0.01,2.73) | 0.30 (0.04,2.34) | -0.23 (-0.74,0.12) | 0.16 (0.01,1.78) | 0.24 (0.04,1.64) | -0.27 (-0.76,0.06) |
| MMF-AZA | CYC HD | 0.40 (0.05,2.15) | 0.47 (0.08,1.83) | -0.11 (-0.37,0.12) | 0.30 (0.06,1.31) | 0.37 (0.08,1.24) | -0.14 (-0.37,0.04) |
| MMF-AZA | CYC-AZA | **0.14 (0.02,0.81)** | **0.26 (0.07,0.84)** | **-0.32 (-0.75,-0.01)** | **0.19 (0.04,0.80)** | **0.28 (0.08,0.83)** | **-0.22 (-0.58,-0.02)** |
| ***New analyses with the removal of observational studies, Mok 2001 and Wang 2008 et al. studies to perform sensitivity analyses based on the exclusion of studies with higher risk of bias.**  **** Included serious infection data rather than sepsis data from Appel 2009 et al. (ALMS study) with all studies including observational data from Mok 2001 and Wang 2008**  **All odds ratios in bold are statistically significant**  High dose (HD) glucocorticoids were defined as one of the following or a similar regimen: (1) prednisone or methylprednisolone 1 gm/m^2^ qd intravenous x 3 at entry, then one dose intravenous q month for 1 year; (2) prednisone 1 mg/kg po qd with a slow taper up to 1 year or longer taper (or unspecified taper in an occasional case).  Glucocorticoids were defined as one of the following or a similar regimen: (1) prednisone 40 mg po qod (every other day) for 8 weeks then taper to 10 mg qd within a year; (2) 60 mg qd for 1-3 months reduced to 20 mg/d by 6 months  CYC, low dose (LD): CYC IV 500 mg q 14 d x 6 doses or a similar regimen  CYC: CYC IV 0.5-1.0 gm/m^2^ q 2month for 1 year or CYC PO 1-4 mg/kg daily for 4 years (standard dose (SD)) or a similar regimen  CYC, HD: CYC IV 0.5-1.0 gm/m^2^ q month x 6-9 months, then q3 months for 0.5-4 years or CYC PO 10 mg/kg daily or a similar regimen  MMF-AZA: MMF followed by AZA  CYC-AZA: CYC followed by AZA | | | | | | | |

**Appendix 9.** Sensitivity analyses: Combining doses CYC and CYC HD into a single group for comparisons vs. main analysis

|  |  | **New Analyses with CYC and CYC HD combined** | | | **Main Analyses (serious infection data only)**** | | |
| --- | --- | --- | --- | --- | --- | --- | --- |
| **Treatment** | **Reference** | **OR (95% CrI)** | **RR (95% CrI)** | **RD (95% Crl)** | **OR (95% CrI)** | **RR (95% CrI)** | **RD (95% Crl)** |
| TAC | Glucocorticoids | **0.30 (0.11,0.77)** | **0.33 (0.13,0.80)** | **-0.09 (-0.16,-0.02)** | **0.36 (0.12,0.98)** | **0.39 (0.14,0.98)** | **-0.08 (-0.15,0.00)** |
| TAC | CYC*** | **0.32 (0.14,0.68)** | **0.35 (0.16,0.71)** | **-0.08 (-0.15,-0.03)** | **0.40 (0.16,0.98)** | **0.44 (0.18,0.98)** | **-0.07 (-0.14,0.00)** |
| TAC | MMF | **0.42 (0.20,0.86)** | **0.45 (0.22,0.88)** | **-0.05 (-0.11,-0.01)** | **0.38 (0.17,0.80)** | **0.41 (0.19,0.82)** | **-0.07 (-0.16,-0.02)** |
| TAC | AZA | **0.34 (0.12,0.87)** | **0.37 (0.15,0.88)** | **-0.08 (-0.17,-0.01)** | **0.32 (0.11,0.87)** | **-0.10 (-0.22,-0.01)** | **-0.10 (-0.22,-0.01)** |
|  |  |  |  |  |  |  |  |
| MMF-AZA | CYC-LD | 0.14 (0.01,1.22) | 0.16 (0.01,1.20) | -0.09 (-0.21,0.02) | 0.38 (0.06,1.86) | 0.44 (0.09,1.69) | -0.10 (-0.38,0.08) |
| MMF-AZA | HD glucocorticoids | 0.06 (0.00,0.92) | 0.08 (0.01,0.93) | -0.20 (-0.66,0.00) | 0.16 (0.01,1.78) | 0.24 (0.04,1.64) | -0.27 (-0.76,0.06) |
| MMF-AZA | CYC HD*** | **0.11 (0.01,0.91)** | **0.13 (0.01,0.92)** | **-0.12 (-0.19,-0.01)** | 0.30 (0.06,1.31) | 0.37 (0.08,1.24) | -0.14 (-0.37,0.04) |
| MMF-AZA | CYC-AZA | **0.14 (0.02,0.76)** | **0.16 (0.02,0.79)** | **-0.09 (-0.24,-0.02)** | **0.19 (0.04,0.80)** | **0.28 (0.08,0.83)** | **-0.22 (-0.58,-0.02)** |
| ***New analyses combined CYC and CYC HD treatment groups**  **** Included serious infection data rather than sepsis data from Appel 2009 et al. (ALMS study) with CYC and CYC HD as separate comparators**  **All odds ratios in bold are statistically significant**  *****This was a combined CYC/CYC HD for the new analysis**  High dose (HD) glucocorticoids were defined as one of the following or a similar regimen: (1) prednisone or methylprednisolone 1 gm/m^2^ qd intravenous x 3 at entry, then one dose intravenous q month for 1 year; (2) prednisone 1 mg/kg po qd with a slow taper up to 1 year or longer taper (or unspecified taper in an occasional case).  Glucocorticoids were defined as one of the following or a similar regimen: (1) prednisone 40 mg po qod for 8 weeks then taper to 10 mg qd within a year; (2) 60 mg qd for 1-3 months reduced to 20 mg/d by 6 months  CYC, low dose (LD): CYC IV 500 mg q 14 d x 6 doses or a similar regimen  CYC: CYC IV 0.5-1.0 gm/m^2^ q 2month for 1 year or CYC PO 1-4 mg/kg daily for 4 yrs (standard dose (SD)) or a similar regimen  CYC, HD: CYC IV 0.5-1.0 gm/m^2^ q month x 6-9 months, then q3 months for 0.5-4 years or CYC PO 10 mg/kg daily or a similar regimen  MMF-AZA: MMF followed by AZA  CYC-AZA: CYC followed by AZA | | | | | | | |

**Appendix 10.** Sensitivity analyses using different priors

|  |  | **Prior=0.292** | **Prior=0.283** | **Prior=0.381** | **Prior=0.40** | **Prior=0.476** | **Prior =0.456** |
| --- | --- | --- | --- | --- | --- | --- | --- |
| **Treatment** | **Reference** | **OR (95% CrI)** | **OR (95% CrI)** | **OR (95% CrI)** | **OR (95% CrI)** | **OR (95% CrI)** | **OR (95% CrI)** |
| CYC | GCC | 0.88(0.46,1.69) | 0.87(0.46,1.69) | 0.88(0.47,1.67) | 0.87(0.46,1.72) | 0.87(0.46,1.65) | 0.88(0.46,1.65) |
| MMF |  | 0.83(0.37,1.86) | 0.84(0.37,1.86) | 0.83(0.38,1.87) | 0.83(0.37,1.85) | 0.82(0.37,1.78) | 0.83(0.38,1.83) |
| AZA |  | 1.04(0.44,2.48) | 1.04(0.44,2.40) | 1.04(0.45,2.41) | 1.03(0.45,2.37) | 1.01(0.44,2.36) | 1.04(0.46,2.39) |
| TAC |  | 0.33(0.12,0.88) | 0.33(0.12,0.87) | 0.33(0.12,0.86) | 0.33(0.12,0.87) | 0.32(0.12,0.86) | 0.33(0.12,0.89) |
| CSA |  | 0.58(0.17,2.01) | 0.59(0.16,2.02) | 0.59(0.17,1.98) | 0.58(0.17,2.02) | 0.56(0.17,1.91) | 0.60(0.17,2.06) |
| CYC LD |  | 1.60(0.50,5.73) | 1.60(0.46,5.75) | 1.63(0.48,5.67) | 1.60(0.48,5.74) | 1.57(0.46,5.33) | 1.59(0.47,5.63) |
| HD GCC |  | 4.19(0.50,37.92) | 4.24(0.48,42.99) | 4.30(0.48,35.04) | 4.33(0.52,38.60) | 3.93(0.46,34.78) | 4.16(0.47,38.70) |
| CYC HD |  | 2.15(0.76,6.73) | 2.15(0.72,6.84) | 2.19(0.75,6.60) | 2.16(0.74,6.68) | 2.11(0.73,6.24) | 2.14(0.74,6.67) |
| AZA HD |  | 1.17(0.09,12.44) | 1.10(0.08,11.64) | 1.18(0.10,11.17) | 1.15(0.09,12.55) | 1.05(0.08,10.66) | 1.12(0.08,12.51) |
| LEF HD |  | 1.84(0.19,20.87) | 1.93(0.19,25.21) | 1.97(0.21,23.31) | 1.91(0.20,24.45) | 1.96(0.19,25.26) | 2.01(0.21,25.11) |
| CYC-AZA |  | 1.07(0.29,3.52) | 1.06(0.28,3.46) | 1.06(0.27,3.50) | 1.06(0.29,3.57) | 1.04(0.29,3.50) | 1.06(0.30,3.54) |
| MMF-AZA |  | 0.15(0.01,1.08) | 0.13(0.01,1.12) | 0.15(0.01,1.14) | 0.14(0.01,1.17) | 0.14(0.01,1.16) | 0.14(0.01,1.15) |
| RTX+MMF |  | 0.85(0.22,3.16) | 0.82(0.22,3.18) | 0.84(0.22,3.04) | 0.82(0.22,3.13) | 0.81(0.22,2.98) | 0.84(0.23,3.14) |
| MMF | CYC | 0.95(0.47,1.84) | 0.95(0.49,1.84) | 0.95(0.49,1.85) | 0.94(0.48,1.84) | 0.93(0.48,1.83) | 0.95(0.49,1.88) |
| AZA |  | 1.18(0.50,2.75) | 1.18(0.54,2.76) | 1.17(0.53,2.70) | 1.17(0.52,2.62) | 1.15(0.51,2.66) | 1.17(0.53,2.69) |
| TAC |  | 0.37(0.15,0.87) | 0.38(0.16,0.87) | 0.37(0.15,0.86) | 0.37(0.15,0.85) | 0.37(0.15,0.86) | 0.38(0.15,0.89) |
| CSA |  | 0.66(0.19,2.19) | 0.66(0.19,2.27) | 0.66(0.20,2.26) | 0.66(0.19,2.24) | 0.64(0.19,2.14) | 0.68(0.21,2.26) |
| CYC LD |  | 1.80(0.60,6.08) | 1.83(0.58,6.14) | 1.84(0.60,6.02) | 1.82(0.58,5.93) | 1.79(0.56,5.90) | 1.80(0.58,6.23) |
| HD GCC |  | 4.70(0.59,41.75) | 4.81(0.58,45.10) | 4.90(0.55,39.44) | 4.90(0.62,42.58) | 4.44(0.53,37.90) | 4.68(0.56,43.80) |
| CYC HD |  | 2.44(0.91,7.12) | 2.47(0.92,7.16) | 2.49(0.93,6.95) | 2.46(0.91,6.88) | 2.40(0.89,6.90) | 2.46(0.89,7.14) |
| AZA HD |  | 1.34(0.10,13.54) | 1.26(0.09,12.62) | 1.34(0.12,12.43) | 1.31(0.10,14.33) | 1.19(0.10,12.11) | 1.29(0.09,13.97) |
| LEF HD |  | 2.08(0.22,23.36) | 2.18(0.23,28.29) | 2.22(0.26,25.41) | 2.17(0.24,27.88) | 2.23(0.23,27.63) | 2.28(0.25,28.02) |
| CYC-AZA |  | 1.21(0.33,3.82) | 1.22(0.34,3.79) | 1.21(0.34,3.76) | 1.20(0.34,3.80) | 1.19(0.34,3.83) | 1.22(0.35,3.89) |
| MMF-AZA |  | 0.17(0.01,1.25) | 0.15(0.01,1.23) | 0.17(0.01,1.24) | 0.16(0.02,1.28) | 0.16(0.01,1.27) | 0.16(0.01,1.30) |
| RTX+MMF |  | 0.96(0.27,3.38) | 0.94(0.27,3.35) | 0.95(0.27,3.26) | 0.94(0.27,3.27) | 0.92(0.26,3.26) | 0.95(0.28,3.36) |
| AZA | MMF | 1.25(0.64,2.46) | 1.24(0.64,2.45) | 1.24(0.64,2.44) | 1.24(0.64,2.41) | 1.24(0.64,2.40) | 1.24(0.65,2.42) |
| TAC |  | 0.40(0.18,0.81) | 0.39(0.18,0.80) | 0.39(0.18,0.79) | 0.40(0.18,0.82) | 0.40(0.18,0.82) | 0.40(0.19,0.80) |
| CSA |  | 0.69(0.21,2.29) | 0.69(0.21,2.36) | 0.70(0.21,2.32) | 0.70(0.22,2.32) | 0.69(0.21,2.18) | 0.71(0.23,2.34) |
| CYC LD |  | 1.92(0.73,5.39) | 1.92(0.71,5.42) | 1.94(0.74,5.39) | 1.93(0.73,5.32) | 1.91(0.72,5.34) | 1.89(0.72,5.46) |
| HD GCC |  | 5.06(0.67,39.43) | 5.07(0.64,44.48) | 5.21(0.62,37.76) | 5.24(0.72,41.25) | 4.78(0.62,36.70) | 4.86(0.64,41.89) |
| CYC HD |  | 2.59(1.16,6.21) | 2.59(1.15,6.08) | 2.61(1.17,6.08) | 2.60(1.17,6.09) | 2.57(1.16,6.15) | 2.56(1.15,6.30) |
| AZA HD |  | 1.43(0.11,13.39) | 1.32(0.10,12.20) | 1.40(0.13,12.24) | 1.38(0.11,14.05) | 1.29(0.11,11.77) | 1.36(0.11,13.66) |
| LEF HD |  | 2.20(0.24,22.50) | 2.30(0.26,27.70) | 2.34(0.29,25.18) | 2.32(0.27,27.05) | 2.39(0.27,27.34) | 2.38(0.29,26.31) |
| CYC-AZA |  | 1.28(0.36,4.08) | 1.27(0.35,4.13) | 1.27(0.36,4.04) | 1.28(0.36,4.01) | 1.28(0.38,4.17) | 1.27(0.37,4.04) |
| MMF-AZA |  | 0.18(0.01,1.33) | 0.16(0.01,1.31) | 0.17(0.02,1.31) | 0.17(0.02,1.34) | 0.17(0.02,1.40) | 0.17(0.01,1.35) |
| RTX+MMF |  | 1.01(0.35,2.89) | 0.99(0.35,2.87) | 1.00(0.35,2.86) | 1.00(0.35,2.88) | 0.99(0.34,2.82) | 1.00(0.35,2.87) |
| TAC | AZA | 0.32(0.12,0.81) | 0.32(0.12,0.81) | 0.31(0.12,0.82) | 0.32(0.12,0.82) | 0.32(0.12,0.82) | 0.32(0.12,0.81) |
| CSA |  | 0.55(0.19,1.59) | 0.56(0.19,1.62) | 0.56(0.20,1.65) | 0.56(0.20,1.67) | 0.55(0.19,1.58) | 0.57(0.20,1.66) |
| CYC LD |  | 1.54(0.51,4.90) | 1.55(0.50,5.05) | 1.56(0.52,5.02) | 1.54(0.51,4.94) | 1.55(0.50,4.87) | 1.53(0.51,5.06) |
| HD GCC |  | 4.08(0.49,32.76) | 4.11(0.47,38.16) | 4.15(0.45,32.82) | 4.23(0.53,36.30) | 3.87(0.46,31.96) | 3.97(0.46,36.42) |
| CYC HD |  | 2.09(0.77,5.87) | 2.08(0.76,5.88) | 2.10(0.78,5.97) | 2.11(0.77,5.80) | 2.08(0.77,5.84) | 2.07(0.77,5.99) |
| AZA HD |  | 1.13(0.09,11.49) | 1.05(0.08,10.60) | 1.11(0.10,10.84) | 1.12(0.09,11.60) | 1.04(0.09,9.96) | 1.10(0.08,11.91) |
| LEF HD |  | 1.77(0.18,19.09) | 1.86(0.20,23.14) | 1.88(0.21,22.24) | 1.89(0.20,22.22) | 1.95(0.20,24.11) | 1.92(0.21,23.12) |
| CYC-AZA |  | 1.03(0.26,3.57) | 1.02(0.26,3.60) | 1.01(0.27,3.53) | 1.02(0.26,3.61) | 1.03(0.27,3.71) | 1.02(0.27,3.63) |
| MMF-AZA |  | 0.14(0.01,1.10) | 0.13(0.01,1.09) | 0.14(0.01,1.11) | 0.13(0.01,1.16) | 0.14(0.01,1.17) | 0.14(0.01,1.18) |
| RTX+MMF |  | 0.81(0.23,2.84) | 0.80(0.23,2.74) | 0.81(0.23,2.71) | 0.81(0.23,2.76) | 0.80(0.23,2.76) | 0.81(0.23,2.82) |
| CSA | TAC | 1.78(0.46,6.97) | 1.79(0.46,7.05) | 1.79(0.46,7.03) | 1.78(0.45,7.18) | 1.74(0.45,6.79) | 1.80(0.48,7.20) |
| CYC LD |  | 4.84(1.48,17.64) | 4.92(1.45,17.46) | 4.95(1.53,17.61) | 4.90(1.46,17.53) | 4.84(1.43,18.19) | 4.79(1.46,17.69) |
| HD GCC |  | 12.83(1.53,119.90) | 12.97(1.45,127.10) | 13.14(1.45,110.40) | 13.25(1.62,122.20) | 12.18(1.43,108.10) | 12.44(1.45,118.30) |
| CYC HD |  | 6.60(2.25,20.50) | 6.65(2.24,20.25) | 6.69(2.31,20.79) | 6.66(2.26,20.67) | 6.49(2.25,21.36) | 6.52(2.25,20.41) |
| AZA HD |  | 3.63(0.26,39.58) | 3.36(0.24,36.15) | 3.59(0.31,34.16) | 3.50(0.26,39.54) | 3.25(0.27,34.29) | 3.46(0.24,37.89) |
| LEF HD |  | 5.63(0.56,62.84) | 5.88(0.61,76.01) | 6.07(0.65,68.59) | 5.92(0.62,74.50) | 6.11(0.59,76.79) | 6.04(0.64,77.72) |
| CYC-AZA |  | 3.25(0.78,12.40) | 3.22(0.80,12.29) | 3.21(0.79,12.51) | 3.23(0.81,12.24) | 3.23(0.81,12.66) | 3.21(0.80,12.46) |
| MMF-AZA |  | 0.44(0.04,3.78) | 0.42(0.03,3.64) | 0.44(0.04,3.76) | 0.43(0.04,3.80) | 0.43(0.04,3.96) | 0.43(0.03,3.71) |
| RTX+MMF |  | 2.57(0.70,9.46) | 2.53(0.70,9.40) | 2.56(0.70,9.31) | 2.53(0.72,9.43) | 2.52(0.69,9.34) | 2.52(0.72,9.34) |
| CYC LD | CSA | 2.78(0.62,12.66) | 2.74(0.61,13.04) | 2.78(0.62,12.89) | 2.76(0.63,12.51) | 2.78(0.64,12.39) | 2.66(0.62,11.96) |
| HD GCC |  | 7.40(0.71,74.14) | 7.23(0.67,83.93) | 7.40(0.65,70.03) | 7.56(0.73,74.76) | 7.02(0.68,68.43) | 6.84(0.66,77.03) |
| CYC HD |  | 3.74(0.92,15.73) | 3.73(0.90,15.50) | 3.73(0.91,15.81) | 3.75(0.90,15.34) | 3.78(0.93,15.75) | 3.62(0.89,14.70) |
| AZA HD |  | 2.04(0.13,25.29) | 1.86(0.12,23.84) | 2.00(0.14,24.47) | 1.98(0.13,25.81) | 1.87(0.13,22.00) | 1.90(0.12,24.61) |
| LEF HD |  | 3.24(0.25,40.62) | 3.37(0.29,46.37) | 3.38(0.31,47.15) | 3.35(0.29,48.48) | 3.52(0.30,51.74) | 3.37(0.29,48.16) |
| CYC-AZA |  | 1.84(0.36,8.72) | 1.81(0.36,8.33) | 1.82(0.34,8.44) | 1.80(0.34,8.75) | 1.85(0.37,9.28) | 1.77(0.36,8.59) |
| MMF-AZA |  | 0.25(0.02,2.54) | 0.23(0.01,2.29) | 0.25(0.02,2.43) | 0.24(0.02,2.53) | 0.25(0.02,2.61) | 0.24(0.02,2.50) |
| RTX+MMF |  | 1.45(0.29,7.03) | 1.43(0.29,6.88) | 1.43(0.29,6.78) | 1.43(0.29,6.88) | 1.46(0.29,6.89) | 1.41(0.29,6.74) |
| HD GCC | CYC LD | 2.62(0.35,20.47) | 2.64(0.36,21.15) | 2.65(0.33,18.35) | 2.72(0.38,20.57) | 2.49(0.34,18.64) | 2.55(0.36,20.62) |
| CYC HD |  | 1.35(0.65,2.71) | 1.36(0.65,2.68) | 1.36(0.66,2.64) | 1.36(0.66,2.70) | 1.35(0.66,2.68) | 1.36(0.66,2.71) |
| AZA HD |  | 0.74(0.06,6.68) | 0.68(0.05,5.91) | 0.72(0.07,5.90) | 0.71(0.06,6.75) | 0.68(0.06,5.63) | 0.70(0.06,6.60) |
| LEF HD |  | 1.15(0.13,11.00) | 1.21(0.14,13.78) | 1.22(0.15,12.32) | 1.21(0.14,12.32) | 1.25(0.14,13.72) | 1.24(0.15,14.03) |
| CYC-AZA |  | 0.66(0.16,2.50) | 0.65(0.15,2.69) | 0.64(0.15,2.46) | 0.66(0.15,2.57) | 0.66(0.16,2.70) | 0.67(0.15,2.62) |
| MMF-AZA |  | 0.09(0.01,0.76) | 0.08(0.01,0.78) | 0.09(0.01,0.75) | 0.09(0.01,0.79) | 0.09(0.01,0.80) | 0.09(0.01,0.79) |
| RTX+MMF |  | 0.53(0.12,2.17) | 0.52(0.11,2.20) | 0.51(0.12,2.11) | 0.52(0.12,2.11) | 0.52(0.12,2.14) | 0.53(0.11,2.16) |
| CYC HD | HD GCC | 0.52(0.08,3.30) | 0.52(0.07,3.27) | 0.51(0.08,3.50) | 0.50(0.08,3.15) | 0.54(0.08,3.47) | 0.53(0.07,3.37) |
| AZA HD |  | 0.28(0.03,2.09) | 0.26(0.02,1.90) | 0.28(0.03,1.89) | 0.26(0.03,1.95) | 0.27(0.03,1.92) | 0.28(0.03,1.94) |
| LEF HD |  | 0.45(0.03,7.06) | 0.47(0.02,8.96) | 0.47(0.03,9.07) | 0.45(0.03,8.20) | 0.50(0.03,9.44) | 0.49(0.03,8.56) |
| CYC-AZA |  | 0.25(0.03,2.36) | 0.25(0.02,2.26) | 0.24(0.03,2.50) | 0.24(0.02,2.32) | 0.27(0.03,2.60) | 0.26(0.03,2.40) |
| MMF-AZA |  | 0.03(0.00,0.56) | 0.03(0.00,0.52) | 0.03(0.00,0.61) | 0.03(0.00,0.54) | 0.03(0.00,0.59) | 0.03(0.00,0.56) |
| RTX+MMF |  | 0.20(0.02,1.93) | 0.20(0.02,2.02) | 0.19(0.02,2.04) | 0.19(0.02,1.83) | 0.21(0.02,2.00) | 0.21(0.02,2.02) |
| AZA HD | CYC HD | 0.55(0.05,4.55) | 0.50(0.05,4.07) | 0.53(0.06,4.04) | 0.53(0.05,4.47) | 0.50(0.05,3.84) | 0.52(0.05,4.40) |
| LEF HD |  | 0.85(0.11,7.34) | 0.88(0.12,9.38) | 0.89(0.13,8.11) | 0.89(0.12,8.61) | 0.92(0.12,9.06) | 0.91(0.13,9.25) |
| CYC-AZA |  | 0.49(0.13,1.68) | 0.49(0.13,1.66) | 0.48(0.13,1.61) | 0.48(0.13,1.65) | 0.49(0.13,1.76) | 0.49(0.13,1.70) |
| MMF-AZA |  | 0.07(0.01,0.54) | 0.06(0.00,0.51) | 0.07(0.01,0.52) | 0.06(0.01,0.53) | 0.07(0.01,0.55) | 0.07(0.01,0.54) |
| RTX+MMF |  | 0.39(0.10,1.47) | 0.38(0.10,1.48) | 0.38(0.10,1.45) | 0.38(0.10,1.41) | 0.39(0.10,1.42) | 0.39(0.10,1.49) |
| LEF HD | AZA HD | 1.60(0.09,38.12) | 1.80(0.09,50.58) | 1.75(0.10,40.69) | 1.76(0.08,42.31) | 1.87(0.10,50.36) | 1.86(0.10,41.31) |
| CYC-AZA |  | 0.89(0.08,13.28) | 0.94(0.08,14.91) | 0.91(0.08,11.38) | 0.92(0.07,12.74) | 0.99(0.09,13.68) | 0.95(0.08,13.08) |
| MMF-AZA |  | 0.12(0.01,2.95) | 0.12(0.00,3.15) | 0.12(0.01,2.62) | 0.12(0.00,2.89) | 0.13(0.01,3.09) | 0.13(0.00,2.84) |
| RTX+MMF |  | 0.72(0.06,11.09) | 0.76(0.06,12.26) | 0.72(0.06,9.82) | 0.73(0.06,10.39) | 0.77(0.07,10.90) | 0.74(0.06,11.45) |
| CYC-AZA | LEF HD | 0.57(0.05,6.16) | **0.54(0.04,6.01)** | **0.53(0.04,5.21)** | **0.55(0.04,5.48)** | **0.52(0.04,5.60)** | 0.52(0.04,5.60) |
| MMF-AZA |  | 0.08(0.00,1.37) | 0.07(0.00,1.35) | 0.07(0.00,1.24) | 0.07(0.00,1.31) | 0.07(0.00,1.31) | 0.07(0.00,1.33) |
| RTX+MMF |  | 0.45(0.04,5.18) | 0.43(0.03,4.76) | 0.42(0.03,4.45) | 0.43(0.03,4.68) | 0.41(0.03,4.63) | 0.42(0.03,4.50) |
| MMF-AZA | CYC-AZA | 0.14(0.02,0.71) | 0.13(0.01,0.69) | 0.14(0.02,0.72) | 0.14(0.02,0.73) | 0.14(0.02,0.73) | 0.14(0.02,0.74) |
| RTX+MMF |  | 0.79(0.16,4.11) | 0.80(0.16,4.05) | 0.80(0.17,4.03) | 0.79(0.17,4.02) | 0.78(0.16,3.84) | 0.79(0.17,4.00) |
| RTX+MMF | MMF-AZA | 5.79(0.59,84.31) | 6.20(0.58,103.80) | 5.81(0.57,78.97) | 6.03(0.57,79.46) | 5.94(0.56,80.22) | 5.89(0.58,81.47) |
|  |  |  |  |  |  |  |  |
|  | **Residual Deviance** | 72.49 vs 71 data point | 72.83 vs. 71 data  points | 72.39 vs. 71 data  points | 72.56 vs. 71 data  points | 72.53 vs. 71 data  points | 72.42 vs. 71 data  points |
|  | **Deviance Information  Criteria** | 325.646 | 326.076 | 325.226 | 325.612 | 325.473 | 325.426 |

CYC, cyclophosphamide; MMF, mycophenolate mofetil; AZA, azathioprine; TAC, tacrolimus; CSA, cyclosporine; GCC, glucocorticoids; LEF, leflunomide; PLASMA, plasmapharesis; RTX, rituximab; HD, high-dose; LD, low-dose

CYC-AZA, CYC followed by AZA

MMF-AZA, MMF followed by AZA

RTX+MMF, RTX combined with MMF

**Appendix 11.** Comparison only significant findings of primary NMA analysis with Sensitivity NMA analysis by year of publication of Contreras study (2004)

|  |  | All Studies | Studies published 2003 or Before | Studies published 2004 or After |
| --- | --- | --- | --- | --- |
| Treatment | Reference | OR (95% CrI) | OR (95% CrI) | OR (95% CrI) |
| TAC | Glucocorticoids | **0.33 (0.12,0.88)** | NA | 0.54 (0.04, 10.01) |
| TAC | CYC | **0.37 (0.15, 0.87)** | NA | 0.41 (0.15,1.12) |
| TAC | MMF | **0.40 (0.18, 0.81)** | NA | **0.38 (0.16,0.84)** |
| TAC | AZA | **0.32 (0.12, 0.81)** | NA | 0.33 (0.10,1.04) |
| MMF-AZA | CYC LD | **0.09 (0.01, 0.76)** | 0.37 (0.02, 7.27) | NA |
| MMF-AZA | HD glucocorticoids | **0.03 (0.00, 0.56)** | 0.08 (0.00, 2.05) | NA |
| MMF-AZA | CYC HD | **0.07 (0.01, 0.54)** | 0.15 (0.01, 2.11) | NA |
| MMF-AZA | CYC-AZA | **0.14 (0.02, 0.71)** | **0.14 (0.02, 0.74)** | NA |
| **Bold numbers indicate the significant results with a p-value <0.05**  OR, odds ratio; CrI, credible interval  NA, not applicable, since there were no studies providing data for these comparisons in these time-periods | | | | |
